# Structural basis of sex pheromone detection in aphids

**DOI:** 10.1038/s41422-026-01267-z

**Published:** 2026-06-22

**Authors:** Zhi Dong, Yidong Wang, Ying Tian, Zeyuan Guan, Minghui Bai, Bo Zhang, Zhongqiang Jia, Jinan Wu, Song Cao, Zhou Gong, Xincheng Zhao, Weihua Ma, Bing Wang, Guirong Wang, Ping Yin

**Affiliations:** 1 National Key Laboratory of Crop Genetic Improvement, College of Bio-X, Hubei Hongshan Laboratory, Huazhong Agricultural University, Wuhan, Hubei China; 2 State Key Laboratory for Biology of Plant Diseases and Insect Pests, Institute of Plant Protection, Chinese Academy of Agricultural Sciences, Beijing, China; 3 Henan International Joint Laboratory of Green Pest Control, College of Plant Protection, Henan Agricultural University, Zhengzhou, Henan China; 4 Shenzhen Branch, Guangdong Laboratory for Lingnan Modern Agriculture, Synthetic Biology Laboratory of the Ministry of Agriculture and Rural Affairs, Agricultural Genomics Institute at Shenzhen, Chinese Academy of Agricultural Sciences, Shenzhen, Guangdong China; 5 School of Life Sciences, Central China Normal University, Wuhan, Hubei China; 6 Innovation Academy for Precision Measurement Science and Technology, Chinese Academy of Sciences, Wuhan, Hubei China

**Keywords:** Cryoelectron microscopy, Ion channel signalling

## Abstract

Sex pheromones play a central role in regulating animal behavior and reproduction. In insects, these signals are perceived through specialized odorant receptors (ORs) that mediate species-specific communication and safeguard genetic integrity. However, the structural basis of sex pheromone detection remains largely unresolved. Here, we identified two ORs in the pea aphid *Acyrthosiphon pisum*, along with the conserved OR co-receptor (Orco), which together mediate recognition of the pheromone components nepetalactone and nepetalactol. Functional assays demonstrated that *Ap*OR21–Orco and *Ap*OR22–Orco specifically respond to nepetalactol and nepetalactone, respectively. Using cryo-electron microscopy, we resolved the structure of the *Ap*OR22–Orco complex in three states — unbound closed, nepetalactone-bound closed, and nepetalactone-bound open — revealing a heterotetrameric ion channel formed by one *Ap*OR22 and three *Ap*Orco subunits. Ligand binding to *Ap*OR22 triggers conformational rearrangements that induce asymmetric pore dilation, thereby enabling ion conduction. Together, these results provide a mechanistic framework for understanding sex pheromone perception in insects and establish a structural foundation for the rational development of environmentally sustainable pest-control strategies.

## Introduction

Sex pheromones are fundamental chemical signals that mediate intraspecific communication, regulating behaviors such as mate attraction, aggression, and reproductive synchronization across diverse taxa.^1^ Species have independently evolved specialized receptors to detect and discriminate sex pheromones emitted by opposite-sex conspecifics.^2,3^ In insects, sex pheromones constitute a primary mode of chemical communication and typically consist of species-specific blends of structurally related molecules.^4^ These pheromones are biosynthesized in glands through enzymatic modification of metabolic precursors.^4^ Most insect pheromone receptors belong to the odorant receptor (OR) family,^5^ whose members require co-expression with the OR co-receptor (Orco) to form functional heteromeric complexes.^6–9^ In moths, OR–Orco complexes mediate the recognition of pheromones such as bombykol,^10^ and in *Drosophila melanogaster*, OR67d in association with Orco detects the male pheromone 11-*cis*-vaccenyl acetate (cVA).^11^

Aphids, which undergo cyclical parthenogenesis, employ sex pheromones during their sexual generation. Oviparous females release pheromones from glandular cells located beneath porous scent plaques on their hind tibiae to attract males.^12–15^ The key pheromone components are the monoterpenoids (+)-(4*aS*,7*S*,7*aR*)-nepetalactone and (–)-(1*R*,4*aS*,7*S*,7*aR*)-nepetalactol, which are ubiquitous in most aphid species, with species-specific activity determined by their ratio.^14,16,17,18^ For example, in the pea aphid *Acyrthosiphon pisum*, the ratio of the two compounds collected using air entrainment is approximately 1:1, whereas in *Megoura viciae*, it ranges from 4:1 to 6:1.^17,18^ The release of sex pheromone in some aphid species also shows significant age dependence, and the ratio of the two compounds changes markedly after pheromone release reaches its peak.^19,20^

Male aphids detect these pheromones via secondary rhinaria on their antennae.^17,18^ Electrophysiological studies have shown that specialized olfactory neurons specifically recognize nepetalactone and nepetalactol, respectively, thereby enabling males to precisely discriminate between different component ratios and thus ensure conspecific recognition.^18,21^ Behavioral studies indicate that a single component alone elicits electrophysiological responses but does not induce full attraction; rather, the two components act synergistically. Nepetalactone primarily mediates long-range attraction, whereas nepetalactol plays a key role in close-range recognition and acts as an aphrodisiac.^20^ Notably, not all aphid sex pheromones conform to this two-component system. For example, the sex pheromone of *Phorodon humuli* consists only of (4*aR*,7*S*,7*aS*)-nepetalactol (likely as a mixture of diastereoisomers) without nepetalactone,^22^ whereas that of *Dysaphis plantaginea* may include a third component, dolichodial.^23^ Such compositional variation contributes to reproductive isolation between species.^21^

Although the biosynthetic pathway of the sex pheromone in *A. pisum* has been fully characterized,^24^ the identity of the pheromone receptors and the molecular mechanisms of pheromone recognition in aphids remain unresolved. Comparative transcriptomic analysis recently revealed that several OR genes, including *Ap*OR17, *Ap*OR21, and *Ap*OR22, are significantly upregulated in males relative to other morphs, making them candidates for sex pheromone detection.^25^ These findings, consistent with those of Robertson et al.,^26^ provide key leads for receptor identification. Owing to its well-characterized chemical communication and rich molecular resources, the pea aphid has become an ideal model for studying the function of sex pheromone receptors.

In this study, we identified two candidate ORs in the pea aphid *A. pisum* that function as sex pheromone receptors, including *Ap*OR22, which responds specifically to nepetalactone. Using cryo-electron microscopy (cryo-EM), we determined the structures of the *Ap*OR22–Orco heterotetramer in three states: nepetalactone-bound open (3.1 Å), nepetalactone-bound closed (2.9 Å), and unbound closed (3.6 Å). Together with biochemical and electrophysiological data, these structures provide mechanistic insights into ligand-specific recognition and channel gating in insect sex pheromone receptors.

## Results

### Identification of sex pheromone receptors in *A. pisum*

**Fig. 1:**
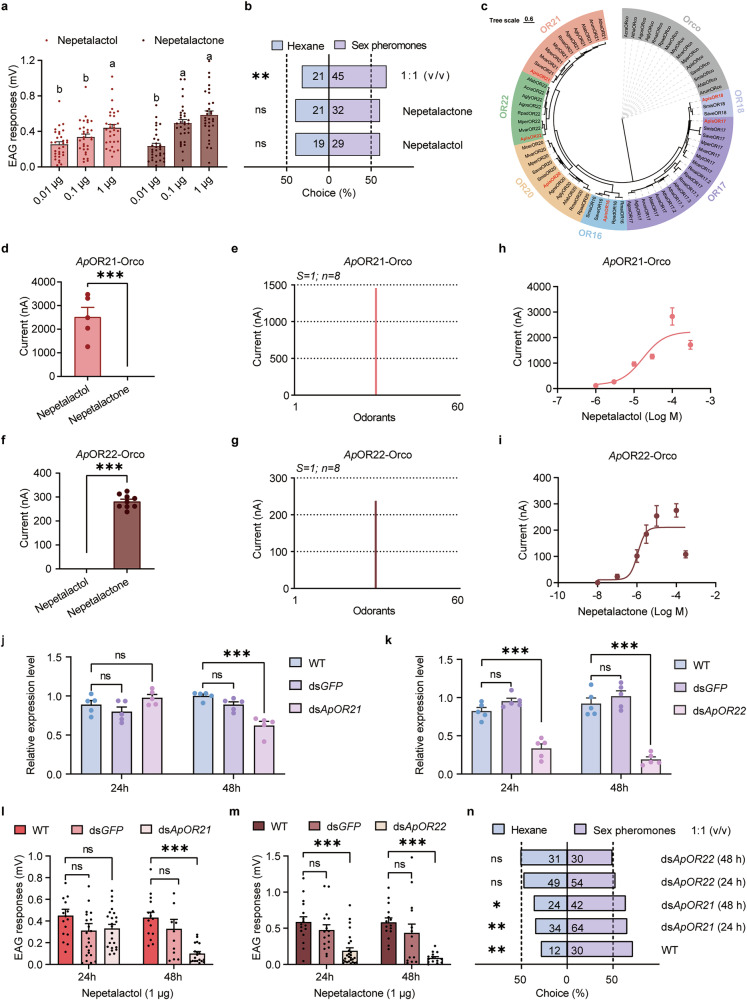
Identification of aphid pheromone receptors. **a** EAG responses of *A. pisum* to nepetalactol and nepetalactone at three doses (0.01–1 μg) (*n* = 31; general linear model followed by Duncan’s multiple range test). Different letters indicate significant differences. Data are shown as means ± SEM. **b** Behavioral choices of* A. pisum* in response to nepetalactol (1 μg), nepetalactone (1 μg), and their 1:1 (v/v) mixture in a T-tube olfactometer. A significant difference in behavioral choice was observed for the 1:1 mixture vs hexane (**P < 0.01; χ^2^ test, χ^2^ = 8.727, *df* = 1, *n* = 66). By contrast, no significant differences were found for nepetalactol or nepetalactone alone vs hexane (ns, *P* > 0.05; χ^2^ test, χ^2^ = 2.083–2.283, *df* = 1, *n* = 48–53). **c** Phylogenetic analysis of candidate sex pheromone receptors from 15 aphid species, encompassing six receptor clades: OR16, OR17, OR18, OR20, OR21, and OR22. *A. pisum* ORs are highlighted in red. **d**, **f** Inward current responses of *Ap*OR21–Orco (**d**, ***P < 0.001, *n* = 5) and *Ap*OR22–Orco (**f**, ***P < 0.001, *n* = 9) to nepetalactol and nepetalactone. **e**, **g** Tuning curves of *Ap*OR21–Orco (**e**) and *Ap*OR22–Orco (**g**) in response to 60 ecologically relevant odorants (*S* = 1, *n* = 8 for each). **h**, **i** Dose-response curves of *Ap*OR21–Orco to nepetalactol (**h**, EC_50_ = 1.708 × 10^−5^ mol/L, *n* = 4) and *Ap*OR22–Orco to nepetalactone (**i**, EC_50_ = 1.096 × 10^−6^ mol/L, *n* = 5). Data are presented as means ± SEM. **j**, **k** Relative expression levels of *Ap*OR21 (**j**) and *Ap*OR22 (**k**) after *dsRNA* infiltration in *A. pisum* (means ± SEM, Student’s *t*-tests, ns, *P* > 0.05; ***P < 0.001, *n* = 5). **l**, **m** EAG responses of *A. pisum* after gene knockdown. Responses to nepetalactol in wild-type, *dsApOR21*-infiltrated, and *dsGFP*-infiltrated *A. pisum* (**l**, means ± SEM, Student’s *t*-tests, ns, *P* > 0.05; ***P < 0.001, *n* = 12–23). Responses to nepetalactone in wild-type, *dsApOR22*-infiltrated, and* dsGFP*-infiltrated *A. pisum* (**m**, means ± SEM, Student’s *t*-tests, ns, *P* > 0.05; ***P < 0.001, *n* = 14–27). **n** Behavioral choices of wild-type and *dsRNA*-infiltrated* A. pisum* between the 1:1 (v/v) mixture of sex pheromone components and hexane in a T-tube olfactometer after 24 h and 48 h (ns, *P* > 0.05; *P < 0.05; **P < 0.01; χ^2^ test, χ^2^ = 0.016–9.184, *df* = 1, *n* = 42–103). For each treatment, 1 μg of the tested compound was used.

Previous studies have shown that the sex pheromone of the pea aphid *A. pisum* consists of nepetalactol and nepetalactone and that a 1:1 mixture of the two components is behaviorally active. To investigate the recognition of these sex pheromones, we first performed electroantennogram (EAG) recordings from male *A. pisum* antennae using the two pheromone components at three doses (0.01–1 μg). Both compounds elicited dose-dependent EAG responses (Fig. 1a). Further behavioral assays showed that neither component alone attracted male aphids; however, the 1:1 mixture elicited significant attraction (*P* < 0.01, Fig. 1b), consistent with previous reports.^17^

To identify the ORs responsible for sex pheromone detection, we prioritized candidates on the basis of three criteria: (1) high expression in male antennae; (2) high predicted binding affinity based on AlphaFold-based screening combined with molecular dynamics-based free energy calculations; and (3) phylogenetic conservation, given that most aphids in the Aphidinae subfamily use nepetalactone and nepetalactol as pheromone components, suggesting that the ORs involved are evolutionarily conserved. Accordingly, we first predicted the ligand-bound structures of all OR–pheromone complexes using AlphaFold and calculated binding free energies (Supplementary information, Table S1). Several ORs — including *Ap*OR3, *Ap*OR4, *Ap*OR13, *Ap*OR16–18, *Ap*OR20–23, *Ap*OR34–39, *Ap*OR49, *Ap*OR67, *Ap*OR79, and *Ap*OR81 — exhibited high binding affinity (≤ –31 kcal/mol), suggesting a potential role in pheromone recognition. Candidate selection was refined using expression data, which revealed male-biased expression of *Ap*OR17–18, *Ap*OR21–22, *Ap*OR26, *Ap*OR31, *Ap*OR33, *Ap*OR72, and *Ap*OR78.^25,26^ Based on this combined analysis, *Ap*OR17–18 and *Ap*OR21–22 emerged as the most likely pheromone receptors.

To assess evolutionary conservation, we examined ORs across Aphidinae species. Phylogenetic analysis of ORs from *A. pisum*, *Aphis glycines*, and *Myzus persicae* revealed that *Ap*OR17–18 and *Ap*OR21–22 belong to a large conserved clade that also contains *Ap*OR16 and *Ap*OR20 (Supplementary information, Figs. S1, S2). A broader phylogenetic reconstruction across 15 Aphidinae species showed that OR20–22 are widely conserved, whereas OR16 and OR18 appear to be lineage-specific. OR17 is broadly present but shows divergence in certain species (Fig. 1c). Together, these structural predictions, expression, and phylogenetic data suggest that the conserved clade containing *Ap*OR16–18 and *Ap*OR20–22 may be functionally important for sex pheromone detection in *A. pisum*.

Functional validation was performed by co-expressing ORs with Orco in *Xenopus* oocytes and recording responses using the two-electrode voltage-clamp (TEVC) method. Among the six tested ORs, *Ap*OR16, *Ap*OR17, *Ap*OR18, and *Ap*OR20 were activated by both nepetalactone and nepetalactol, with *Ap*OR16, *Ap*OR17, and *Ap*OR20 showing significantly stronger responses to nepetalactol (Supplementary information, Fig. S3). By contrast, *Ap*OR21 responded exclusively to nepetalactol, and *Ap*OR22 was activated only by nepetalactone (Fig. 1d, f). For subsequent in vitro functional assays, we selected 60 ecologically relevant odorants and systematically tested the ligand-binding profiles of *Ap*OR21 and *Ap*OR22 (Supplementary information, Table S2). The results showed that *Ap*OR21 is narrowly tuned to nepetalactol, whereas *Ap*OR22 is narrowly tuned to nepetalactone (Fig. 1d–g). Dose-response curves confirmed that *Ap*OR21 and *Ap*OR22 recognize their respective ligands in a concentration-dependent manner (Fig. 1h, i). These results indicate that all six candidate ORs are involved in pheromone detection in *A. pisum*, with *Ap*OR21 and *Ap*OR22 exhibiting specific recognition.

We next performed in vivo functional validation experiments for *Ap*OR21 and *Ap*OR22. After knockdown of *ApOR21* for 48 h (*P* < 0.001, Fig. 1j), males showed a significantly reduced electrophysiological response to nepetalactol (*P* < 0.001, Fig. 1l), accompanied by decreased attraction to the 1:1 (v/v) pheromone mixture in behavioral assays (Fig. 1n). Knockdown of *ApOR22* for both 24 h and 48 h (*P* < 0.001, Fig. 1k) significantly reduced the male electrophysiological response to nepetalactone (*P* < 0.001, Fig. 1m) and completely abolished the attraction effect (Fig. 1n). These findings collectively demonstrate that *Ap*OR21 and *Ap*OR22 play critical roles in the recognition of nepetalactol and nepetalactone, respectively, and that both are involved in the detection of sex pheromone components and the resulting behavioral responses in male *A. pisum*.

### Structural determination of the *Ap*OR22–Orco complex

To examine the molecular mechanism of pheromone recognition, we used cryo-EM to resolve OR–Orco complexes. Six candidate ORs (*Ap*OR16–18 and *Ap*OR20–22) were co-expressed with *Ap*Orco in HEK293 cells. All ORs were modified with an N-terminal Twin-Strep-superfolder green fluorescent protein (sfGFP) tandem tag, whereas *Ap*Orco featured an N-terminal 3× Flag epitope. *Ap*OR22–Orco exhibited the highest expression levels (often associated with relatively greater stability and homogeneity) and was selected for structural analysis because of its critical role in pheromone recognition (Supplementary information, Fig. S4a). However, this complex exhibited instability and heterogeneity, likely due to an N-terminal flexible loop predicted by AlphaFold 2 (Supplementary information, Fig. S4a, b). Sequence alignment of the six ORs revealed divergence in this region, prompting the design of a 33-residue N-terminal truncation mutant (OR22Δ33, hereafter referred to as *Ap*OR22) (Supplementary information, Figs. S2, S4c). The truncation produced a homogeneous, stable complex suitable for cryo-EM while retaining functional responsiveness to nepetalactone, as verified by TEVC assays (Supplementary information, Fig. S4d, e).

To obtain the ligand-bound structure, purified *Ap*OR22–Orco was incubated with 0.7 mM nepetalactone before vitrification. However, reconstruction yielded only a ligand-free, gate-closed channel at 3.6 Å resolution, likely owing to insufficient ligand occupancy. Increasing the incubation concentration to 10 mM generated two ligand-bound conformations: a nepetalactone-bound open state at 3.1 Å and a nepetalactone-bound closed state at 2.9 Å, representing distinct conformational intermediates (Supplementary information, Fig. S5). These maps enabled near-complete atomic model building, with the exception of the flexible intracellular N-termini — including the affinity tags — and portions of the S4–S5 loops in both the OR and Orco subunits (Supplementary information, Figs. S5, S6).

### Architecture of the *Ap*OR22–Orco complex

**Fig. 2:**
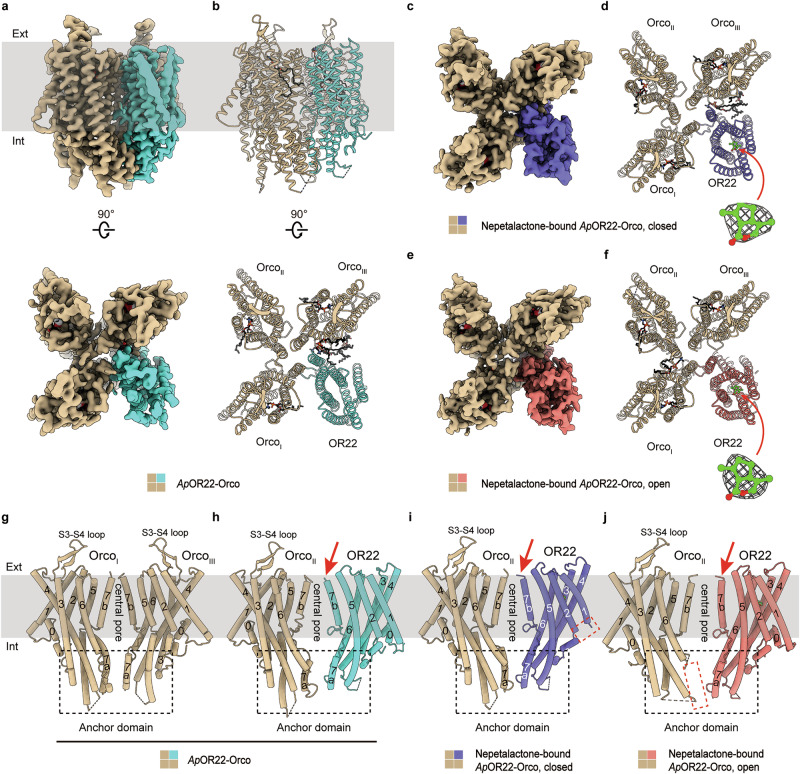
Structures of the *Ap*OR22–Orco complex. **a**–**f** EM density maps and overall structures of *Ap*OR22–Orco in the unbound closed (**a**, **b**), nepetalactone-bound closed (**c**, **d**), and nepetalactone-bound open (**e**, **f**) states. Panels show side (top) and extracellular (bottom) views for the unbound closed state (**a**, **b**); only extracellular views are shown for ligand-bound states (**c**–**f**). Maps were generated in ChimeraX at contour levels of 0.15 (**a**), 0.10 (**c**), and 0.15 (**e**). Membrane boundaries are indicated by gray boxes (**a**, **b**). Red arrows mark ligand-binding pockets; close-up views of ligand densities are shown in (**d**, **f**). Lipids are depicted as black sticks. Ext, extracellular; Int, intracellular. **g** Lateral view of the Orco_I_ subunit and the diagonal Orco_Ⅲ_ subunit in the unbound closed state. **h**–**j** Lateral views of *Ap*OR22 with the diagonal Orco_II_ subunit in the three structural states. *Ap*Orco subunits are shown in tan; *Ap*OR22 is shown in cyan (unbound closed), light purple (nepetalactone-bound closed), or salmon (nepetalactone-bound open). Red dashed boxes highlight unresolved regions.

Across all three conformational states, *Ap*OR22–Orco adopts a heterotetrameric assembly of one *Ap*OR22 subunit with three *Ap*Orco subunits (Fig. 2a–f; Supplementary information, Fig. S5). Three-dimensional (3D) classification in our cryo-EM analysis revealed multiple low-resolution classes, but only the 3:1 configuration (Orco:OR) yielded maps at sufficient resolution for atomic modeling, consistent with previous reports (Supplementary information, Figs. S5, S7a).^27,28^ Each subunit contains seven transmembrane helices (S1–S7) and a short re-entrant N-terminal helix (S0). The S7 helices from all four subunits form the central pore domain, whereas S1–S6 project radially outward (Fig. 2g–j). The S7 helix is divided into S7a and S7b, with the C-terminal S7b segments converging to form the channel gate. Portions of helices S4–S7 extend intracellularly, forming the anchor domain (Fig. 2g–j). In both nepetalactone-bound states, unassigned densities within the *Ap*OR22 transmembrane region matched the size of a bound ligand (Fig. 2d, f). Some regions, including partial S6 and S7 helices of *Ap*Orco in the open state and the S0 helix of *Ap*OR22 in the closed state, were unresolved owing to local flexibility (Fig. 2i, j).

**Fig. 3:**
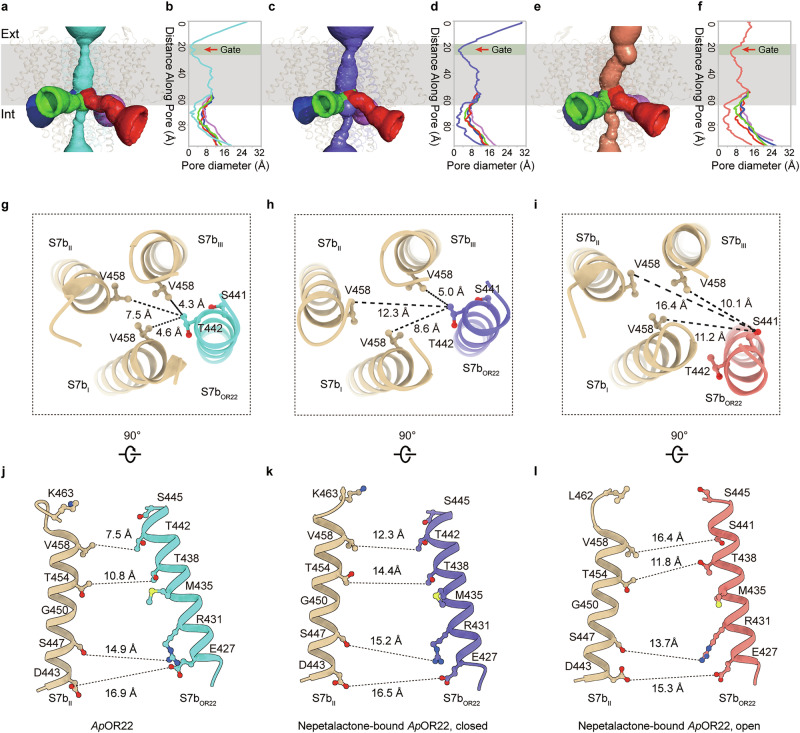
The ion-conducting pore of *Ap*OR22–Orco. **a**–**f** Channel pores and calculated pore diameters of *Ap*OR22–Orco in the unbound closed (**a**, **b**), nepetalactone-bound closed (**c**, **d**), and nepetalactone-bound open (**e**, **f**) states. The ion-conducting pore radius was calculated with the HOLE program. Ext, extracellular; Int, intracellular. **g**–**i** Distances between gate residues in the unbound closed (**g**), nepetalactone-bound closed (**h**), and nepetalactone-bound open (**i**) states. **j**–**l** Residues lining the central pore of *Ap*OR22–Orco in the unbound closed (**j**), nepetalactone-bound closed (**k**), and nepetalactone-bound open (**l**) conformations.

The *Ap*OR22–Orco complex functions as an ion channel comprising a central pore and four lateral conduits between subunits, with the central pore opening toward the cytoplasm (Fig. 3a–f). Comparison of pore diameters revealed substantial conformational changes at the channel gate. The narrowest constrictions were 0.8 Å and 2.0 Å in the unbound and nepetalactone-bound closed states, respectively, but expanded to 5.5 Å in the nepetalactone-bound open state (Fig. 3a–f). Considering that the effective radii of hydrated cations (e.g., Na^+^, K^+^, and Ca^2+^) typically range from 1.7 Å to 6.6 Å, this opening is consistent with previously resolved OR/gustatory receptor (GR) structures, in which open-state pore sizes generally range from 3 Å to 6 Å.^27–33^ These dimensions are sufficient for ion permeation, which likely involves the passage of ions in a partially hydrated state. Channel gating is controlled by residues V458 (*Ap*Orco S7b) and S441/T442 (*Ap*OR22 S7b), as shown by changes in inter-residue distances. In the unbound state, T442–V458 distances were 4.6 Å, 7.5 Å, and 4.3 Å (Fig. 3g, j). In the ligand-bound closed state, these distances expanded to 8.6 Å, 12.3 Å, and 5.0 Å (Fig. 3h, k). In the ligand-bound open state, T442 was replaced by S441, with S441–V458 distances of 11.2 Å, 16.4 Å, and 10.1 Å (Fig. 3i, l), confirming the closed vs open configurations.

Interestingly, the positions of gate residues in *Ap*OR22–Orco differ from those of previously characterized OR–Orco complexes.^27,28^ In ligand-bound *Ap*OR5–Orco, *Aa*OR10–*Ab*Orco, and *Ag*OR28–*Ab*Orco, gate residues are located at the 21st position of S7b in Orco and the 20th position in OR (S357 in *Ap*OR5, R372 in *Aa*OR10, and T394 in *Ag*OR28) (Supplementary information, Fig. S7b). By contrast, in the open-state *Ap*OR22–Orco complex, gate residues are positioned at the 17th and 16th positions of S7b in Orco and OR22, respectively. This shift reflects the shorter S7b segment in *Ap*OR22 (by ≥ 3 residues), which places the gate one helical turn lower than in other ORs. Consequently, the C-terminal end of Orco following V458 fails to form a regular helix owing to the absence of stabilizing van der Waals contacts with the S7b helix of *Ap*OR22 (Fig. 3j–l; Supplementary information, Fig. S7b).

### Ligand binding pocket of *Ap*OR22

**Fig. 4:**
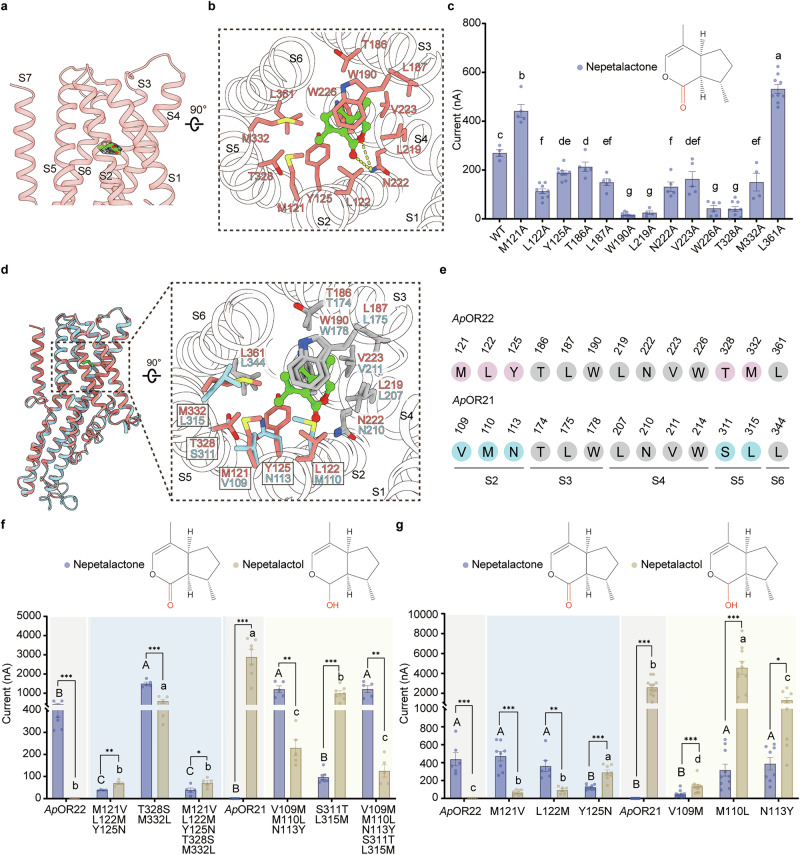
Odorant binding of *Ap*OR22–Orco. **a** Lateral view of *Ap*OR22 in the nepetalactone-bound open state. **b** Close-up view of the ligand-binding pocket, with interacting residues highlighted. Yellow dashed lines represent hydrogen bonds. **c** Functional responses of wild-type *Ap*OR22 and mutant variants to nepetalactone. Significant differences, determined by one-way ANOVA with Duncan’s test, are indicated by different lowercase letters (α = 0.05). Error bars represent SEM (*n* = 4–9). **d** Structural comparison of *Ap*OR22 in the nepetalactone-bound open state and modeled *Ap*OR21. Close-up views of the ligand-binding pockets show interacting residues in *Ap*OR22 and corresponding residues in *Ap*OR21. Black boxes highlight divergent residues. **e** Sequence alignment of proposed ligand-binding residues in *Ap*OR22 and *Ap*OR21. Identical residues are colored gray; divergent residues are colored pink for *Ap*OR22 and blue for *Ap*OR21. **f**, **g** Functional responses of wild-type *Ap*OR22,* Ap*OR21, and mutant variants to nepetalactone and nepetalactol. Comparisons between ligands were evaluated using Student’s *t*-tests (***P < 0.001; **P < 0.01; *P < 0.05). Significant differences, determined by one-way ANOVA with Duncan’s test (α = 0.05), in responses to nepetalactone and nepetalactol are indicated by different uppercase and lowercase letters, respectively. Error bars represent SEM (*n* = 4–12).

In the ligand-bound *Ap*OR22–Orco structures, we identified a binding pocket located in the central region of the transmembrane domain, enclosed by the S2–S6 helices (Fig. 4a). Within this pocket, an additional density was observed that fit the shape of a ligand but could not be attributed to protein. We hypothesized that this density corresponds to nepetalactone (Fig. 4a). Molecular docking within this pocket produced three ranked configurations of nepetalactone (Supplementary information, Fig. S8a–d). The lowest-energy configuration (rank 1) contained nine nearly identical poses (Supplementary information, Fig. S8b). This pose was selected to refine the nepetalactone-bound *Ap*OR22 model (Supplementary information, Fig. S8e, f). Given the similarity of the pocket and ligand density between the nepetalactone-bound open and closed states, we used the open-state pose to refine the closed-state model. In both states, nepetalactone occupied a nearly identical position (Supplementary information, Fig. S8g–i).

In the nepetalactone-bound open state, the ligand interacts with 13 residues, including nine hydrophobic and four polar amino acids (Fig. 4b). Notably, residue N222 forms two hydrogen bonds with the ligand’s polar C=O group, stabilizing its orientation (Fig. 4b). To test the functional importance of these residues, we generated alanine substitutions and measured ligand responsiveness using TEVC assays. Eleven of the 13 mutants showed markedly reduced responses compared with wild-type *Ap*OR22, whereas two mutants (M121A and L361A) displayed enhanced sensitivity (Fig. 4c). Molecular dynamics simulations revealed that the T328 and M332 residues on the S5 helix, a region crucial for receptor activation, shifted slightly toward the ligand compared with the wild-type structure. This subtle rearrangement offers a potential mechanism for the enhanced response currents observed (Supplementary information, Fig. S9). These findings confirm that the identified residues form the nepetalactone-binding interface. Comparison of the nepetalactone-bound *Ap*OR22 structure with previously resolved ligand-bound OR structures (*Ap*OR5, *Aa*OR10, and *Ag*OR28) revealed that ligands consistently bound in equivalent positions, fully enclosed within the receptor. However, the pocket size varied depending on ligand dimensions (Supplementary information, Fig. S7c).

### Ligand specificity determinants in *Ap*OR22

Electrophysiological recordings demonstrated distinct ligand preferences between *Ap*OR22 and *Ap*OR21, despite ~60% sequence identity (Fig. 1d–i; Supplementary information, Fig. S2). To investigate the structural basis for this difference, we modeled *Ap*OR21 using the nepetalactone-bound *Ap*OR22 open structure as a template and compared the two structures (Fig. 4d). The overall architecture of the ligand-binding pocket was conserved, but five residues diverged: M121/L122/Y125 (S2 helix) and T328/M332 (S5 helix) in *Ap*OR22 vs V109/M110/N113 (S2 helix) and S311/L315 (S5 helix) in *Ap*OR21 (Fig. 4d, e). We hypothesized that these divergent residues dictate ligand selectivity.

To test this possibility, we engineered chimeric receptors by reciprocally exchanging (1) the S2 triad (*Ap*OR22^M121V/L122M/Y125N^ and *Ap*OR21^V109M/M110L/N113Y^), (2) the S5 pair (*Ap*OR22^T328S/M332L^ and *Ap*OR21^S311T/L315M^), and (3) both regions simultaneously. Functional assays revealed that the *Ap*OR22^M121V/L122M/Y125N^ variant exhibited greater responsiveness to nepetalactol than to nepetalactone, although its overall sensitivity was low. Conversely, *Ap*OR21^V109M/M110L/N113Y^ displayed the opposite preference, favoring nepetalactone (Fig. 4f). Substitutions in the S5 helix modulated activation strength: *Ap*OR22^T328S/M332L^ showed enhanced responsiveness to nepetalactone and partial activation by nepetalactol, whereas *Ap*OR21^S311T/L315M^ showed reduced nepetalactol sensitivity but gained weak nepetalactone responsiveness (Fig. 4f). Dual swaps largely reproduced the phenotypes of the S2 triad swaps, underscoring the dominant role of S2 residues in defining ligand specificity (Fig. 4f).

As *Ap*OR22^M121V/L122M/Y125N^ displayed weak responses to both ligands, we generated single-point mutants (*Ap*OR22^M121V^, *Ap*OR22^L122M^, and *Ap*OR22^Y125N^) to parse their contributions. Of these, only *Ap*OR22^Y125N^ exhibited a stronger response to nepetalactol than to nepetalactone (Fig. 4g). Similarly, testing *Ap*OR21 (*Ap*OR21^V109M^, *Ap*OR21^M110L^, and *Ap*OR21^N113Y^) revealed that *Ap*OR21^N113Y^ responded robustly to nepetalactone, mirroring wild-type *Ap*OR22, but showed reduced responsiveness to nepetalactol compared with wild-type *Ap*OR21 (Fig. 4g). These results suggest that Y125 in *Ap*OR22 and the corresponding N113 in *Ap*OR21 are critical determinants of ligand preference. The inversion of ligand preference in *Ap*OR21^V109M/M110L/N113Y^ further suggests that the S2 triad fine-tunes specificity. Nevertheless, the fact that none of the swap variants could completely reverse the ligand specificity indicates that specificity arises from the collective and often cooperative action of multiple binding-pocket residues rather than a single site.

### Activation mechanism of the *Ap*OR22–Orco complex

**Fig. 5:**
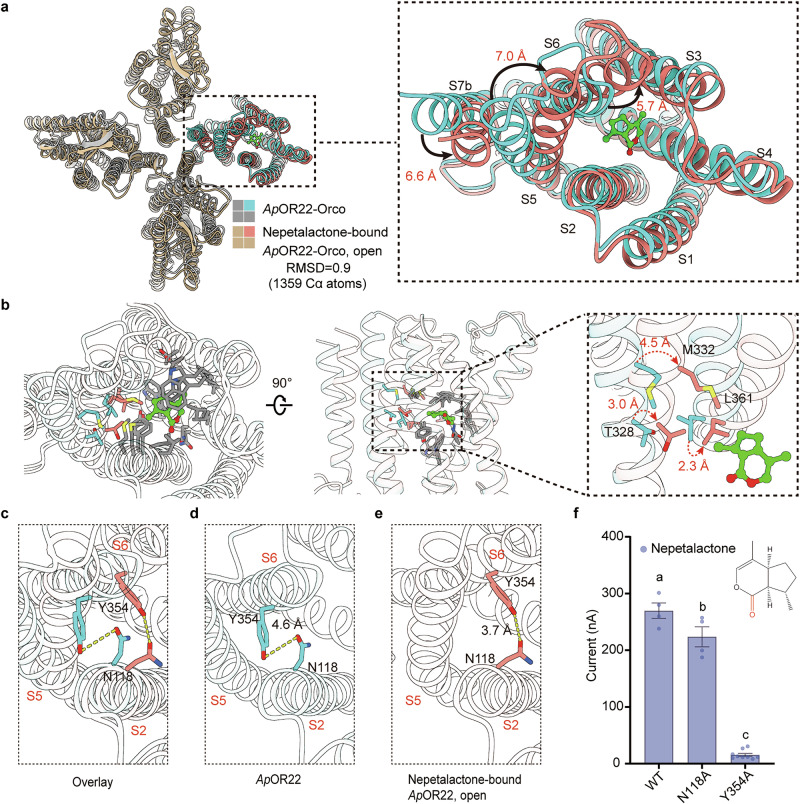
The activation mechanism of *Ap*OR22–Orco. **a** Superimposition of *Ap*OR22–Orco structures in the unbound closed and nepetalactone-bound open states, viewed extracellularly. The expanded view highlights conformational shifts, with black arrows indicating helix rotation. **b** Close-up view of the nepetalactone-binding pocket, showing residue differences between the unbound closed and nepetalactone-bound open states. Red dashed arrows indicate residue rotation. Nepetalactone is shown as a lime ball-and-stick model; cyan and salmon sticks represent residues in the unbound closed and nepetalactone-bound open states, respectively. **c**–**e** A hydrogen bond between N118 and Y354 is observed in the nepetalactone-bound open state but absent in the unbound closed state. Yellow dashed lines denote the N118–Y354 distance. **f** Electrophysiological responses of wild-type *Ap*OR22 and two mutant variants to nepetalactone. Mutant responses are compared with those of the wild type. Significant differences, determined by one-way ANOVA with Duncan’s test, are indicated by different lowercase letters (α = 0.05). Error bars represent SEM (*n* = 4–9).

To determine how ligand binding activates the *Ap*OR22–Orco complex, we compared the structures of the unbound and ligand-bound open states. Overall, the complex displayed minimal global differences, with a root-mean-square deviation (RMSD) of only 0.9 Å (Fig. 5a). Structural rearrangements were confined primarily to *Ap*OR22. Helices S1–S4 remained largely unchanged, whereas S5, S6, and S7 near the extracellular side underwent marked shifts. Specifically, helices S5, S6, and S7 rotated toward the ligand by ~7.0 Å, 5.7 Å, and 6.6 Å, respectively (Fig. 5a). The outward movement of S7 relative to the pore axis widened the channel, resembling the asymmetric gating mechanism reported in other OR–Orco complexes.^27,28^

Within the ligand-binding site, only residues T328, M332, and L361 shifted significantly — by 3.0 Å, 4.5 Å, and 2.3 Å, respectively — toward the ligand in the open state, whereas other binding residues remained largely unchanged (Fig. 5b). T328 and M332 lie on S5, whereas L361 resides on S6. Their displacement deflected the S5 and S6 helices, which, through hydrophobic coupling with adjacent residues (Supplementary information, Fig. S10a–c), subsequently drove the repositioning of S7. In addition, a unique hydrogen bond between Y354 and N118 formed exclusively in the open state (Fig. 5c–e). Mutational disruption of this bond, particularly substitution of Y354, markedly reduced ligand responsiveness (Fig. 5f). Together, these findings indicate that ligand binding induces coordinated rearrangements of S5–S7, coupled with the Y354–N118 interaction, to trigger channel opening.

Comparison of the ligand-bound closed state with the unbound and open conformations further clarified this gating process. Relative to the unbound state, S5, S6, and S7 in the closed state shifted toward the ligand by 4.3 Å, 4.1 Å, and 3.8 Å, respectively (Supplementary information, Fig. S10d). Compared with the open state, however, the same helices were displaced 3.3 Å, 2.3 Å, and 7.0 Å away from their open-state positions (Supplementary information, Fig. S10f). Thus, in the closed conformation, S5 and S6 occupy an intermediate position, whereas S7 moves closer to the Orco subunit than in the unbound state, collectively maintaining a sealed gate.

At the ligand-binding site, residues T328, M332, and L361 shifted 1.9 Å, 2.5 Å, and 1.8 Å toward the ligand in the closed state relative to the unbound conformation (Supplementary information, Fig. S10e). However, compared with the open state, T328 and M332 exhibited smaller displacements of 1.2 Å and 2.1 Å, respectively (Supplementary information, Fig. S10g). Importantly, the repositioning of Y354 increased its distance from N118 to 5.0 Å, abolishing the stabilizing hydrogen bond (Supplementary information, Fig. S10h, i). These alterations weakened ligand interactions involving T328 and M332, preventing S5 and S6 from pivoting sufficiently to pull S7 away from the gate. Consequently, the pore remained closed.

## Discussion

**Fig. 6:**
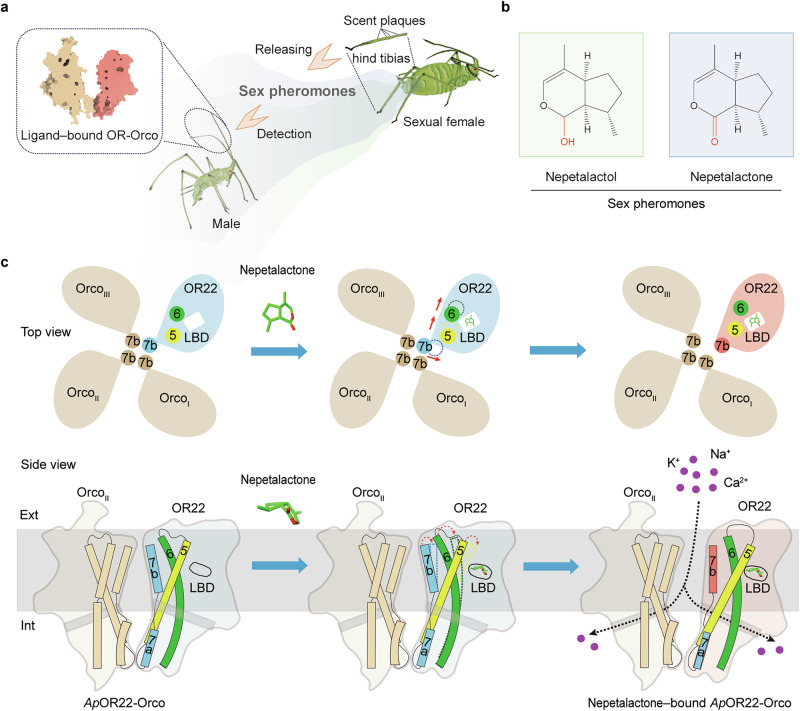
Model of aphid sex pheromone recognition and the *Ap*OR22–Orco activation mechanism. **a** Schematic of sex pheromone recognition in the pea aphid. Scent plaques located on the hind tibiae of oviparous sexual females release pheromones composed primarily of nepetalactol and nepetalactone. These compounds are detected by ORs expressed in the sensory neurons of male antennae, which guide mating behavior. **b** Schematic representation of sex pheromone components in the pea aphid. **c** Activation model of the *Ap*OR22–Orco complex. Binding of nepetalactone to the ligand-binding domain of *Ap*OR22 induces a conformational change, leading to deflection of the S5 and S6 helices. This helical rearrangement triggers the rotation of S7b, ultimately opening the channel pore for ion influx. Dashed circles highlight the S5–S7b helices in the ligand-bound open state, with red arrows indicating the direction of deflection. LBD, ligand-binding domain.

Sex pheromones are central to insect reproduction and survival, offering promising avenues for sustainable pest management.^1^ The sex pheromones nepetalactone and nepetalactol have been definitively identified in aphids (Fig. 6a, b),^17,18^ but the receptors responsible for their detection have not been characterized, limiting both mechanistic insight and practical application. Although previous studies have suggested that morphology-specific chemosensory genes might mediate pheromone perception,^25,26^ functional validation has been lacking. Here, we identified two sex pheromone receptors (PRs) in the pea aphid *A. pisum* and resolved the structures of one receptor, *Ap*OR22, in three distinct states, providing detailed insight into the molecular basis of insect sex pheromone detection (Fig. 6c). Given the high conservation of aphid sex pheromones, these findings not only advance our understanding of odorant-driven signal transduction but also provide a foundation for the rational design of agrochemicals to improve sustainable pest management.

Comparison of the *Ap*OR22–Orco complex with the previously reported *Ap*OR5–Orco heterocomplex reveals both conserved principles and distinct adaptations in the function of insect olfactory receptors.^27^ Both complexes adopt a conserved 1 OR:3 Orco heterotetrameric stoichiometry and share a similar overall topology, including the general location of the ligand-binding pocket. However, although both are narrowly tuned, their strict ligand specificity arises from unique binding-pocket architectures tailored to their respective ligands. *Ap*OR22 selectively binds the cyclic monoterpene nepetalactone, which interacts broadly with residues located primarily on the surrounding helices (S2–S6), forming an extensive yet compact interface. By contrast, *Ap*OR5 recognizes the longer, acyclic terpenoid geranyl acetate, which not only engages transmembrane helices but also induces ordering of, and interaction with, the S3–S4 loop.

In terms of the gating mechanism, both complexes exhibit ligand-induced conformational changes that drive outward movement of the OR subunit’s S7b helix, leading to asymmetric pore opening. However, the specific structural rearrangements differ. In the *Ap*OR22–Orco complex, nepetalactone binding induces a cooperative deflection of the S5 and S6 helices and triggers a unique open-state-specific hydrogen bond (Y354–N118) essential for effective activation. Furthermore, the S7b segment of *Ap*OR22 is shorter than that of other ORs (e.g., *Ap*OR5), which shifts its gate-forming residues toward the cytosolic side by one helical turn. These subtle yet functionally important variations demonstrate that, while operating within a conserved activation framework, individual OR subunits have evolved specialized conformational pathways to precisely tune their responses to different ligand classes. This highlights distinct strategies for achieving high ligand selectivity and efficient signal transduction, distinguishing the detection of sex pheromones from that of other general odorants.

*Ap*OR22 and *Ap*OR21 specifically recognize nepetalactone and nepetalactol, respectively. Although their ligand-binding pockets differ by only five amino acid residues, our residue-swap experiments failed to fully reverse their ligand preferences (Fig. 4f, g). These results demonstrate that ligand specificity in these receptors is not solely a binary function of a few defined binding-pocket residues. Although residues such as Y125 (*Ap*OR22) and V109/M110/N113 (*Ap*OR21) are critical determinants, the overall selectivity landscape is likely shaped by the broader structural and chemical context of the entire binding pocket. This context differs inherently between *Ap*OR21 and *Ap*OR22, despite their overall similarity. Subtle differences in the arrangement of other residues surrounding the binding site, or allosteric influences from more distant regions, may fine-tune the binding energetics and gating coupling efficiency differently in each receptor background. Therefore, a symmetric “residue swap” may not be sufficient to fully overcome these inherent structural differences and produce a complete functional swap.

In light of the recently reported *Bm*GR9 structure,^30^ our structural and functional analysis of *Ap*OR22 reveals both shared and distinct principles governing ligand-gated activation and ligand specificity in insect chemoreceptors. Although both receptors use movement of the S5 helix and an S5–S7 bridge to couple ligand binding to pore opening, the architectural details differ. The ligand-binding site in *Ap*OR22 is positioned deeper within the transmembrane bundle than the more superficial site in *Bm*GR9.^30^ This fundamental difference alters the role of the critical S5 bridge residues. In *Bm*GR9, the bridge residue F333 contacts the sugar ligand, directly linking selectivity to gating. By contrast, although *Ap*OR22 residues T328 and M332 undergo conformational changes crucial for ligand binding and receptor activation, *Ap*OR22 uses a more distributed network of residues within the binding pocket to fine-tune ligand recognition. This suggests that although the overall activation mechanism may be conserved, the specific molecular levers for achieving ligand specificity have evolved differently in these receptors. Future structural studies on diverse ORs and GRs will be essential to map the full spectrum of ligand recognition strategies in these important receptor families.

## Materials and methods

### Gene annotation and phylogenetic analysis

Phylogenetic analysis of ORs was performed across three previously characterized aphid species (*A. pisum*, *A. glycines*, and *M. persicae*).^26,34^ Protein sequences were aligned with MAFFT (v7.487) and trimmed with trimAl (v1.4.rev15) using default parameters.^35,36^ Trees were reconstructed with IQ-TREE (v2.1.4-beta) using “-m MFP -bb 1000” and visualized with ChiPlot.^37,38^ Candidate PRs were comprehensively annotated in 15 aphid species using *A. pisum* PRs as queries. Sequences of PRs and Orcos from these species are provided in Supplementary information, Data S1. Initial predictions were filtered using the hidden Markov model PF02949.23 (7tm_6) with BITACORA (v1.4) in genome mode and an e-value cutoff of 1e−5.^39^ A PR-specific hidden Markov model was then built with HMMER (v3.3.2).^40^ Iterative refinement with HMMER yielded a high-confidence PR annotation set, which was used to reconstruct the PR family phylogeny following the same pipeline.

### Construct design and protein expression

Full-length coding sequences of *Ap*OR22 and *Ap*Orco were amplified from *A. pisum*. Site-directed mutagenesis and amino-terminal truncations of *Ap*OR22 were performed using Fusion PCR. For heterologous expression in HEK293F cells (Invitrogen), gene fragments were subcloned into a modified pMlink vector.^41^ Constructs for wild-type, truncated, and mutant *Ap*OR22 carried an N-terminal Twin-Strep-tag (WSHPQFEKGGGARGGSGGGSWSHPQFEK), superfolder GFP (sfGFP),^42^ and a TEV protease site. *Ap*Orco was fused to an N-terminal triple Flag tag (DYKDDDDKGDYKDDDDKIDYKDDDDK). For *Xenopus laevis* oocyte assays, *Ap*OR22 (wild-type, truncated, or mutant), *Ap*OR16, *Ap*OR17, *Ap*OR18, *Ap*OR20, *Ap*OR21, and *Ap*Orco were cloned into pT7Ts as described previously.^43^

HEK293F cells were cultured in Union-293 medium (Union-Biotech, Shanghai, China) at 37 °C with 5% CO₂ and 110 rpm agitation. For transfection, pMlink plasmids encoding *Ap*OR22 and *Ap*Orco were delivered with 4 kDa linear polyethylenimine (PEI, Polysciences). After 60 h, cells were collected by centrifugation at 2000× *g* for 15 min, washed with PBS, flash-frozen in liquid nitrogen, and stored at −80 °C until use.

### TEVC electrophysiological recordings

Heterologous expression in *X. laevis* oocytes followed standard protocols, including cRNA synthesis, enzymatic defolliculation, co-injection of OR and Orco cRNAs, and incubation in supplemented Ringer’s buffer.^43^ Electrophysiological recordings were performed using the TEVC method as described previously.^44^ Whole-cell currents were recorded with an OC-725C amplifier (Warner Instruments, Hamden, CT, USA) at a holding potential of −80 mV. Oocytes were stimulated with the sex pheromone components nepetalactone and nepetalactol, as well as 58 other ecologically relevant odorants (Supplementary information, Table S2). Wild-type and mutant *Ap*ORs co-expressed with *Ap*Orco were analyzed. Between stimulations, oocytes were washed thoroughly with 1× Ringer’s buffer to restore baseline currents. Data were acquired with a Digidata 1440 A system and analyzed using pClamp 10.0 (Axon Instruments, Union City, CA, USA). Dose-response relationships were fitted using GraphPad Prism 9.3 (GraphPad Software, San Diego, CA, USA).

### RT-qPCR

TRIzol reagent (Invitrogen, USA) was used to extract total RNA from wild-type and RNAi-treated aphids. First-strand cDNA was synthesized with a RevertAid First Strand cDNA Synthesis kit (Thermo Fisher Scientific, USA). Quantitative real-time PCR was performed on a QuantStudio 6 Flex Real-Time PCR System (Thermo Fisher Scientific, USA) using GoTaq qPCR Master Mix (Promega, USA). Gene expression was normalized to that of two reference genes, *Ap*Tubulin and *Ap*Actin, and relative fold changes were calculated using the 2^−ΔΔCt^ method. Primer sequences are listed in Supplementary information, Table S4.

### EAG recordings

Antennae were excised at the base, the tips were cut, and the antennae were then mounted in glass electrodes filled with 0.1 M KCl. Test solutions (10 μL) containing either an odorant or solvent were pipetted onto filter paper strips, which were placed inside Pasteur pipettes. Odorant stocks (1 M) were diluted with paraffin oil. A CS-55 stimulus controller (Syntech, Germany) provided a humidified main air flow of 30 mL/s. Stimuli were delivered as 0.2-s pulses at 10 mL/s, with a 30-s inter-stimulus interval. Signals were amplified with a 10× AC/DC preamplifier (Syntech) and recorded with an IDAC4-USB controller (Syntech). Data acquisition and analysis were performed using Syntech EAG software. EAG responses to pheromone stimuli were measured in wild-type and RNAi-treated aphids.

### Behavioral experiments

A glass T-tube olfactometer (1.5-cm internal diameter, 1-cm stem length, and 20-cm arm length) was used for behavioral assays. Filter paper strips loaded with 10 μL of sex pheromone (either nepetalactone, nepetalactol, or their 1:1 mixture) or 10 μL of hexane, as a solvent control, were randomly placed at the distal ends of the two arms. For each treatment, the total dose of the tested compound was 1 μg. Individual aphids were introduced into the tube, and their movements were observed. A choice was recorded when an aphid walked beyond one-third of the length of either arm and remained there for at least 30 s. Each treatment was replicated 42–103 times, with one aphid per replicate.

### Protein purification

Frozen HEK293F pellets were thawed at room temperature and resuspended in buffer A (100 mM Tris-HCl, pH 8.0, 150 mM NaCl, and 1 mM EDTA). Proteins were extracted with 1% (w/v) lauryl maltose neopentyl glycol (LMNG, Anatrace) for 2 h at 4 °C. The lysate was centrifuged at 20,000× *g* for 1 h at 4 °C to remove debris. The supernatant was incubated with Strep-Tactin resin (IBA) for 1 h at 4 °C, washed with buffer W1 (100 mM Tris-HCl, pH 8.0, 150 mM NaCl, 1 mM EDTA, and 0.02% GDN), and eluted with W1 containing 50 mM d-biotin (Sangon Biotech). The eluate was passed over anti-Flag G1 resin (GenScript) for 1 h at 4 °C, washed with buffer W2 (50 mM Tris-HCl, pH 8.0, 150 mM NaCl, and 0.02% GDN), and eluted with W2 containing 500 µg/mL 3× Flag peptide. Samples were concentrated and loaded onto a Superose 6 Increase column (GE Healthcare) pre-equilibrated with buffer B (25 mM Tris-HCl, pH 8.0, 150 mM NaCl, and 0.02% GDN). Peak fractions corresponding to the *Ap*OR22–Orco complex were pooled and concentrated to ~6.0 mg/mL for immediate cryo-EM grid preparation. For ligand-bound complexes, peak fractions were incubated with (+)-(4*aS*,7*S*,7*aR*)-nepetalactone (RTC, CAS: 21651-62-7) dissolved in DMSO at a final concentration of 0.7 mM or 10 mM for 1 h at 4 °C prior to grid freezing.

### Cryo-EM grid preparation and data acquisition

Aliquots (3.5 µL) of purified *Ap*OR22–Orco complex (with or without nepetalactone incubation) were applied to glow-discharged holey carbon grids (Quantifoil Cu R1.2/1.3, 300 mesh). Grids were blotted on a Vitrobot Mark IV (Thermo Fisher Scientific) for 3.5 s at 100% humidity and 8 °C, then plunge-frozen into liquid ethane cooled by liquid nitrogen. Grids were screened and imaged on a 300 kV Titan Krios transmission electron microscope (Thermo Fisher Scientific) equipped with a Gatan K3 detector and GIF Quantum energy filter. Micrographs of the unbound state were recorded at a nominal magnification of 105,000× (pixel size 0.82 Å). Image stacks of 40 movie frames were collected with a defocus range of −1.0 to −2.0 µm, an exposure time of 1.9 s, and a total dose of 50 e^−^/Å.^2^ For the ligand-bound state, micrographs were recorded at 105,000× (pixel size 0.824 Å); image stacks of 40 frames were collected with a defocus range of −1.3 to −1.8 µm, an exposure time of 2.16 s, and a total dose of 50 e^−^/Å.^2^

### Cryo-EM data processing

The data-processing workflow is summarized in Supplementary information, Fig. S4. For the *Ap*OR22–Orco dataset, 3058 image stacks were collected. All stacks were motion-corrected with MotionCor2.^45^ A total of 2,832,005 particles were automatically picked from 2808 micrographs using cryoSPARC blob picker^46^ and Topaz (v0.2.3).^47^ After iterative two-dimensional (2D) classification, 514,649 high-quality particles were retained for multiple rounds of 3D classification in cryoSPARC.^46^ Particles from the best class were refined with non-uniform and local refinement procedures, yielding a reconstruction at an estimated global resolution of 3.6 Å by gold-standard Fourier shell correlation (FSC).^48^ Local resolution variation was estimated using Reamap (v1.1.4).^49^

For the nepetalactone-bound *Ap*OR22–Orco dataset, 7331 movies were collected and motion-corrected with MotionCor2. From 7173 selected micrographs, 2,429,970 particles were automatically picked using cryoSPARC blob picker^46^ and Topaz (v0.2.3).^47^ Following several rounds of 2D and 3D classification, 391,342 particles were retained and separated into five conformational classes. One class (82,222 particles) was refined to produce the nepetalactone-bound open reconstruction (reported resolution 3.1 Å), and the remaining four classes (309,120 particles combined) were merged and refined to yield the nepetalactone-bound closed reconstruction (reported resolution 2.9 Å), as assessed by gold-standard FSC.^49,50^ Local resolution maps were generated using Reamap (v1.1.4).^49^

### Model building and refinement

An initial backbone trace of the *Ap*OR22–Orco complex was generated de novo using DeepTracer (v1.0).^48^ AlphaFold 2-predicted models of *Ap*OR22 and *Ap*Orco^51^ were docked into this backbone to guide chain assignment and register. Models were iteratively adjusted in Coot,^52^ and lipid-like densities were modeled as phosphatidylcholines, consistent with membrane composition.^53^ Real-space refinement against the maps was performed in PHENIX (phenix.real_space_refine) with secondary-structure and geometry restraints. Model quality was evaluated using MolProbity scores,^54^ Ramachandran statistics, and EMRinger.^55^ Structural figures were prepared in ChimeraX (https://www.cgl.ucsf.edu/chimerax/) and PyMOL (https://pymol.org/2/). RMSDs between structures were calculated using PyMOL. The ion-conducting pore radius was calculated using the HOLE program.^56^

### Homology modeling

The full-length coding sequences of *Ap*OR21 were used for modeling. The nepetalactone-bound open-state cryo-EM structure of *Ap*OR22 served as the template. Multiple sequence alignments were generated with MUSCLE^57^ and manually refined. For each OR, 200 models were built with MODELLER 9.3.^58^ Models were ranked by the MODELLER objective function and evaluated with QMEANBrane.^59^ Stereochemical quality was assessed by Ramachandran analysis using MolProbity (http://molprobity.biochem.duke.edu/index.php).

### Molecular docking analysis

Putative ligand pockets in each OR model were defined by the position of nepetalactone in the *Ap*OR22 structure. 3D structures of *cis*/*trans*-nepetalactol (CAS: 109215-55-6) and *cis*/*trans*-nepetalactone (CAS: 21651-62-7) were retrieved from PubChem. Docking was performed with AutoDock 4.2.6,^60^ generating 256 poses per OR–ligand pair. Poses were clustered by RMSD, and the lowest-energy conformation from the largest cluster was selected as the representative binding mode. Protein–ligand interactions were profiled with PLIP,^61^ and docking results were visualized in PyMOL (v2.1).

### Structural prediction and energy minimization

Structural predictions were performed using AlphaFold 3^62^ for 87 distinct OR proteins in complex with two small molecules: nepetalactone and nepetalactol. Each complex subsequently underwent conformational optimization using AMBER 20^63^ to eliminate force-field mismatches and sterically unreasonable conformations. The protein components were described using the AMBER ff19SB force field,^64^ and the small molecules were parameterized using the General Amber Force Field (GAFF).^65^ Binding free energies between the ligands and proteins were calculated for each optimized conformation using the Molecular Mechanics/Generalized Born Surface Area (MM/GBSA) approach.^66^

### Molecular dynamics simulations

For wild-type *Ap*OR22 and mutants (L361A and M121A) complexed with nepetalactone, molecular dynamics simulations were performed using AMBER 20 to evaluate the effects of the mutations on protein–ligand interactions. Initial systems were constructed using the CHARMM-GUI web server^67^ by embedding the proteins into a POPC (1-palmitoyl-2-oleoyl-glycero-3-phosphocholine) bilayer composed of 120 lipid molecules, solvated with a 20 Å water layer containing 0.15 M NaCl. Protein orientations relative to the membrane were determined using the OPM database (aligned via the PPM 2.0 web server),^68^ resulting in system dimensions of approximately 75 × 75 × 140 Å^3^. The proteins were described using the AMBER ff99SB force field, the ligand was treated with GAFF, and the lipid21 force field^69^ was used for POPC. Following energy minimization, the systems were gradually heated to 303 K and subjected to a 500-ps equilibration period at this temperature. Production runs were subsequently performed at 303 K and 1 bar for 1 μs with a 2-fs time step. Three independent replicates were performed for each system, yielding 5000 snapshots per trajectory. RMSDs of the protein and ligand were analyzed using the CPPTRAJ module in AMBER 22, and binding free energies were calculated using the MM/GBSA method.

## Supplementary information

Supplementary information, Fig. S1

Supplementary information, Fig. S2

Supplementary information, Fig. S3

Supplementary information, Fig. S4

Supplementary information, Fig. S5

Supplementary information, Fig. S6

Supplementary information, Fig. S7

Supplementary information, Fig. S8

Supplementary information, Fig. S9

Supplementary information, Fig. S10

Supplementary information, Table. S1

Supplementary information, Table. S2

Supplementary information, Table. S3

Supplementary information, Table. S4

Supplementary information, Data. S1

## Data Availability

The atomic coordinates and EM density for the reported structures of the unbound *Ap*OR22–Orco (PDB: 9WPF; EMDB: EMD-66141) and nepetalactone-bound *Ap*OR22–Orco complex in the open (PDB: 9WPG; EMDB: EMD-66142) and closed (PDB: 9WPE; EMDB: EMD-66140) states have been deposited in the Protein Data Bank (PDB, www.rcsb.org) and the Electron Microscopy Data Bank (EMDB), respectively. All other data necessary to evaluate the conclusions of this paper are present in the main text and/or the supplementary materials.

## References

[1] Gomez-Diaz, C. & Benton, R. The joy of sex pheromones. *EMBO Rep.* **14**, 874–883 (2013). 24030282 10.1038/embor.2013.140 PMC3807217

[2] Touhara, K. & Vosshall, L. B. Sensing odorants and pheromones with chemosensory receptors. *Annu. Rev. Physiol.* **71**, 307–332 (2009). 19575682 10.1146/annurev.physiol.010908.163209

[3] Silbering, A. F. & Benton, R. Ionotropic and metabotropic mechanisms in chemoreception: ‘chance or design’? *EMBO Rep.* **11**, 173–179 (2010). 20111052 10.1038/embor.2010.8 PMC2838705

[4] Jurenka, R. A. In Insect Pheromone Biochemistry and Molecular Biology (Second Edition) (eds. Gary J. B. & Richard G. V.) 13–88 Academic Press, (2021).

[5] Fleischer, J. & Krieger, J. Insect pheromone receptors - key elements in sensing intraspecific chemical signals. *Front. Cell Neurosci.* **12**, 425 (2018). 30515079 10.3389/fncel.2018.00425 PMC6255830

[6] Sato, K. et al. Insect olfactory receptors are heteromeric ligand-gated ion channels. *Nature* **452**, 1002–1006 (2008). 18408712 10.1038/nature06850

[7] Wicher, D. et al. *Drosophila* odorant receptors are both ligand-gated and cyclic-nucleotide-activated cation channels. *Nature* **452**, 1007–1011 (2008). 18408711 10.1038/nature06861

[8] Liu, Q. et al. Deletion of the *Bombyx mori* odorant receptor co-receptor (BmOrco) impairs olfactory sensitivity in silkworms. *Insect Biochem. Mol. Biol.* **86**, 58–67 (2017). 28577927 10.1016/j.ibmb.2017.05.007

[9] Fan, X. B. et al. Mutagenesis of the odorant receptor co-receptor (Orco) reveals severe olfactory defects in the crop pest moth *Helicoverpa armigera*. *BMC Biol.* **20**, 214 (2022). 36175945 10.1186/s12915-022-01411-2 PMC9524114

[10] Sakurai, T. et al. Identification and functional characterization of a sex pheromone receptor in the silkmoth *Bombyx mori*. *Proc. Natl. Acad. Sci. USA* **101**, 16653–16658 (2004). 15545611 10.1073/pnas.0407596101 PMC528734

[11] Kurtovic, A., Widmer, A. & Dickson, B. J. A single class of olfactory neurons mediates behavioural responses to a* Drosophila* sex pheromone. *Nature* **446**, 542–546 (2007). 17392786 10.1038/nature05672

[12] Pettersson, J. An aphid sex attractant. II. Histological, ethological and comparative studies. Vol. 2 81–93 Entomologica Scandinavica, (1971).

[13] Harrington, R. A comparison of the external morphology of ‘scent plaques’ on the hind tibiae of oviparous aphids (Homoptera: Aphididae). *Syst. Entomol.* **10**, 135–144 (1985).

[14] Pickett, J. A., Wadhams, L. J., Woodcock, C. M. & Hardie, J. The chemical ecology of aphids. *Annu. Rev. Entomol.* **37**, 67–90 (1992).

[15] Murano, K., Ogawa, K., Kaji, T. & Miura, T. Pheromone gland development and monoterpenoid synthesis specific to oviparous females in the pea aphid. *Zool. Lett.* **4**, 9 (2018). 10.1186/s40851-018-0092-0 PMC5946545 29780614

[16] Birkett, M. A. & Pickett, J. A. Aphid sex pheromones: from discovery to commercial production. *Phytochemistry* **62**, 651–656 (2003). 12620315 10.1016/s0031-9422(02)00568-x

[17] Dawson, G. W. et al. Identification of an aphid sex pheromone. *Nature* **325**, 614–616 (1987).

[18] Dawson, G. W. et al. Aphid semiochemicals - A review, and recent advances on the sex pheromone. *J. Chem. Ecol.* **16**, 3019–3030 (1990). 24263293 10.1007/BF00979609

[19] Marsh, D. Sex pheromone in the aphid* Megoura viciae*. *Nat. New Biol.* **238**, 31–32 (1972). 18663846 10.1038/newbio238031a0

[20] Hardie, J. et al. Aphid sex pheromone components: Age-dependent release by females and species-specific male response. *Chemoecology* **1**, 63–68 (1990).

[21] Guldemond, J. A., Dixon, A. F. G., Pickett, J. A., Wadhams, L. J. & Woodcock, C. M. Specificity of sex pheromones, the role of host plant odour in the olfactory attraction of males, and mate recognition in the aphid *Cryptomyzus*. *Physiol. Entomol.* **18**, 137–143 (1993).

[22] Campbell, C. A. et al. Sex attractant pheromone of damson-hop aphid *Phorodon humuli* (Homoptera, aphididae). *J. Chem. Ecol.* **16**, 3455–3465 (1990). 24263441 10.1007/BF00982110

[23] Dewhirst, S. Y. et al. Dolichodial: a new aphid sex pheromone component? *J. Chem. Ecol.* **34**, 1575–1583 (2008). 19023626 10.1007/s10886-008-9561-9

[24] Kollner, T. G. et al. Biosynthesis of iridoid sex pheromones in aphids. *Proc. Natl. Acad. Sci. USA* **119**, e2211254119 (2022). 36227916 10.1073/pnas.2211254119 PMC9586269

[25] Purandare, S. R. & Brisson, J. A. Divergent chemosensory gene expression accompanies ecological specialisation of pea aphid morphs. *Ecol. Entomol.* **45**, 364–368 (2020).

[26] Robertson, H. M., Robertson, E. C. N., Walden, K. K. O., Enders, L. S. & Miller, N. J. The chemoreceptors and odorant binding proteins of the soybean and pea aphids. *Insect Biochem. Mol. Biol.* **105**, 69–78 (2019). 30654011 10.1016/j.ibmb.2019.01.005

[27] Wang, Y. et al. Structural basis for odorant recognition of the insect odorant receptor OR-Orco heterocomplex. *Science* **384**, 1453–1460 (2024). 38870272 10.1126/science.adn6881

[28] Zhao, J., Chen, A. Q., Ryu, J. & Del Marmol, J. Structural basis of odor sensing by insect heteromeric odorant receptors. *Science* **384**, 1460–1467 (2024). 38870275 10.1126/science.adn6384 PMC11235583

[29] Ma, D. et al. Structural basis for sugar perception by *Drosophila* gustatory receptors. *Science* **383**, eadj2609 (2024). 38305684 10.1126/science.adj2609

[30] Gomes, J. V. et al. The molecular basis of sugar detection by an insect taste receptor. *Nature* **629**, 228–234 (2024). 38447670 10.1038/s41586-024-07255-w PMC11062906

[31] Del Marmol, J., Yedlin, M. A. & Ruta, V. The structural basis of odorant recognition in insect olfactory receptors. *Nature* **597**, 126–131 (2021). 34349260 10.1038/s41586-021-03794-8 PMC8410599

[32] Yang, Z.-H. The size and structure of selected hydrated ions and implications for ion channel selectivity. *RSC Adv.* **5**, 1213–1219 (2015).

[33] Barger, J. P. & Dillon, P. F. Near-membrane electric field calcium ion dehydration. *Cell Calcium* **60**, 415–422 (2016). 27683058 10.1016/j.ceca.2016.09.006

[34] Liu, J. et al. Identification and tissue expression profiles of odorant receptor genes in the green peach aphid *Myzus persicae*. *Insects* **13**, 398 (2022). 10.3390/insects13050398 PMC9147661 35621734

[35] Capella-Gutierrez, S., Silla-Martinez, J. M. & Gabaldon, T. trimAl: a tool for automated alignment trimming in large-scale phylogenetic analyses. *Bioinformatics* **25**, 1972–1973 (2009). 19505945 10.1093/bioinformatics/btp348 PMC2712344

[36] Katoh, K. & Standley, D. M. MAFFT multiple sequence alignment software version 7: improvements in performance and usability. *Mol. Biol. Evol.* **30**, 772–780 (2013). 23329690 10.1093/molbev/mst010 PMC3603318

[37] Minh, B. Q. et al. IQ-TREE 2: New models and efficient methods for phylogenetic inference in the genomic era. *Mol. Biol. Evol.* **37**, 1530–1534 (2020). 32011700 10.1093/molbev/msaa015 PMC7182206

[38] Xie, J. et al. Tree Visualization By One Table (tvBOT): a web application for visualizing, modifying and annotating phylogenetic trees. *Nucleic Acids Res.* **51**, W587–W592 (2023). 37144476 10.1093/nar/gkad359 PMC10320113

[39] Vizueta, J., Sanchez-Gracia, A. & Rozas, J. Bitacora: A comprehensive tool for the identification and annotation of gene families in genome assemblies. *Mol. Ecol. Resour.* **20**, 1445–1452 (2020). 32492257 10.1111/1755-0998.13202

[40] Potter, S. C. et al. HMMER web server: 2018 update. *Nucleic Acids Res.* **46**, W200–W204 (2018). 29905871 10.1093/nar/gky448 PMC6030962

[41] Lu, P. et al. Three-dimensional structure of human gamma-secretase. *Nature* **512**, 166–170 (2014). 25043039 10.1038/nature13567 PMC4134323

[42] Pedelacq, J. D., Cabantous, S., Tran, T., Terwilliger, T. C. & Waldo, G. S. Engineering and characterization of a superfolder green fluorescent protein. *Nat. Biotechnol.* **24**, 79–88 (2006). 16369541 10.1038/nbt1172

[43] Zhang, R. et al. Molecular basis of alarm pheromone detection in aphids. *Curr. Biol.* **27**, 55–61 (2017). 27916525 10.1016/j.cub.2016.10.013

[44] Wang, B., Cao, S., Liu, W. & Wang, G. Effect of OBPs on the response of olfactory receptors. *Methods Enzymol.* **642**, 279–300 (2020). 32828257 10.1016/bs.mie.2020.04.065

[45] Zheng, S. Q. et al. MotionCor2: anisotropic correction of beam-induced motion for improved cryo-electron microscopy. *Nat. Methods* **14**, 331–332 (2017). 28250466 10.1038/nmeth.4193 PMC5494038

[46] Punjani, A., Rubinstein, J. L., Fleet, D. J. & Brubaker, M. A. cryoSPARC: algorithms for rapid unsupervised cryo-EM structure determination. *Nat. Methods* **14**, 290–296 (2017). 28165473 10.1038/nmeth.4169

[47] Bepler, T. et al. Positive-unlabeled convolutional neural networks for particle picking in cryo-electron micrographs. *Nat. Methods* **16**, 1153–1160 (2019). 31591578 10.1038/s41592-019-0575-8 PMC6858545

[48] Pfab, J., Phan, N. M. & Si, D. DeepTracer for fast de novo cryo-EM protein structure modeling and special studies on CoV-related complexes. *Proc. Natl. Acad. Sci. USA* **118**, e2017525118 (2021). 10.1073/pnas.2017525118 PMC7812826 33361332

[49] Kucukelbir, A., Sigworth, F. J. & Tagare, H. D. Quantifying the local resolution of cryo-EM density maps. *Nat. Methods* **11**, 63–65 (2014). 24213166 10.1038/nmeth.2727 PMC3903095

[50] Chen, S. et al. High-resolution noise substitution to measure overfitting and validate resolution in 3D structure determination by single particle electron cryomicroscopy. *Ultramicroscopy* **135**, 24–35 (2013). 23872039 10.1016/j.ultramic.2013.06.004 PMC3834153

[51] Jumper, J. et al. Highly accurate protein structure prediction with AlphaFold. *Nature* **596**, 583–589 (2021). 34265844 10.1038/s41586-021-03819-2 PMC8371605

[52] Brown, A. et al. Tools for macromolecular model building and refinement into electron cryo-microscopy reconstructions. *Acta Crystallogr. D Biol. Crystallogr.* **71**, 136–153 (2015). 25615868 10.1107/S1399004714021683 PMC4304694

[53] Vance, J. E. Phospholipid synthesis and transport in mammalian cells. *Traffic* **16**, 1–18 (2015). 25243850 10.1111/tra.12230

[54] Williams, C. J. et al. MolProbity: more and better reference data for improved all-atom structure validation. *Protein Sci.* **27**, 293–315 (2018). 29067766 10.1002/pro.3330 PMC5734394

[55] Barad, B. A. et al. EMRinger: side chain-directed model and map validation for 3D cryo-electron microscopy. *Nat. Methods* **12**, 943–946 (2015). 26280328 10.1038/nmeth.3541 PMC4589481

[56] Smart, O. S., Neduvelil, J. G., Wang, X., Wallace, B. A. & Sansom, M. S. HOLE: a program for the analysis of the pore dimensions of ion channel structural models. *J. Mol. Graph.* **14**, 354–360, 376 (1996). 9195488 10.1016/s0263-7855(97)00009-x

[57] Madeira, F. et al. The EMBL-EBI Job Dispatcher sequence analysis tools framework in 2024. *Nucleic Acids Res.* **52**, W521–W525 (2024). 38597606 10.1093/nar/gkae241 PMC11223882

[58] Webb, B. & Sali, A. Comparative protein structure modeling using MODELLER. *Curr. Protoc. Protein Sci.* **86**, 2.9.1–2.9.37 (2016). 27801516 10.1002/cpps.20

[59] Studer, G., Biasini, M. & Schwede, T. Assessing the local structural quality of transmembrane protein models using statistical potentials (QMEANBrane). *Bioinformatics* **30**, i505–i511 (2014). 25161240 10.1093/bioinformatics/btu457 PMC4147910

[60] Morris, G. M. et al. AutoDock4 and AutoDockTools4: Automated docking with selective receptor flexibility. *J. Comput. Chem.* **30**, 2785–2791 (2009). 19399780 10.1002/jcc.21256 PMC2760638

[61] Adasme, M. F. et al. PLIP 2021: expanding the scope of the protein-ligand interaction profiler to DNA and RNA. *Nucleic Acids Res.* **49**, W530–W534 (2021). 33950214 10.1093/nar/gkab294 PMC8262720

[62] Abramson, J. et al. Accurate structure prediction of biomolecular interactions with AlphaFold 3. *Nature* **630**, 493–500 (2024). 38718835 10.1038/s41586-024-07487-w PMC11168924

[63] Case, D. A. et al. AMBER 2020. University of California, San Francisco (2020).

[64] Tian, C. et al. ff19SB: Amino-acid-specific protein backbone parameters trained against quantum mechanics energy surfaces in solution. *J. Chem. Theory Comput.* **16**, 528–552 (2020). 31714766 10.1021/acs.jctc.9b00591 PMC13071887

[65] Wang, J., Wolf, R. M., Caldwell, J. W., Kollman, P. A. & Case, D. A. Development and testing of a general amber force field. *J. Comput. Chem.* **25**, 1157–1174 (2004). 15116359 10.1002/jcc.20035

[66] Miller, B. R. et al. MMPBSA.py: an efficient program for end-state free energy calculations. *J. Chem. Theory Comput.* **8**, 3314–3321 (2012). 26605738 10.1021/ct300418h

[67] Jo, S., Kim, T., Iyer, V. G. & Im, W. CHARMM-GUI: a web-based graphical user interface for CHARMM. *J. Comput. Chem.* **29**, 1859–1865 (2008). 18351591 10.1002/jcc.20945

[68] Lomize, M. A., Pogozheva, I. D., Joo, H., Mosberg, H. I. & Lomize, A. L. OPM database and PPM web server: resources for positioning of proteins in membranes. *Nucleic Acids Res.* **40**, D370–D376 (2012). 21890895 10.1093/nar/gkr703 PMC3245162

[69] Dickson, C. J., Walker, R. C. & Gould, I. R. Lipid21: complex lipid membrane simulations with AMBER. *J. Chem. Theory Comput.* **18**, 1726–1736 (2022). 35113553 10.1021/acs.jctc.1c01217 PMC9007451

