# Supplementary material for: Structural basis of sex pheromone detection in aphids
Source: Cell Res. 2026 Jun 22;36(8):582–94. doi: 10.1038/s41422-026-01267-z (PMC13424144; doi:10.1038/s41422-026-01267-z)
Supplement: Supplementary file 3 — Supplementary information, Fig. S3 [file 41422_2026_1267_MOESM3_ESM.pdf]

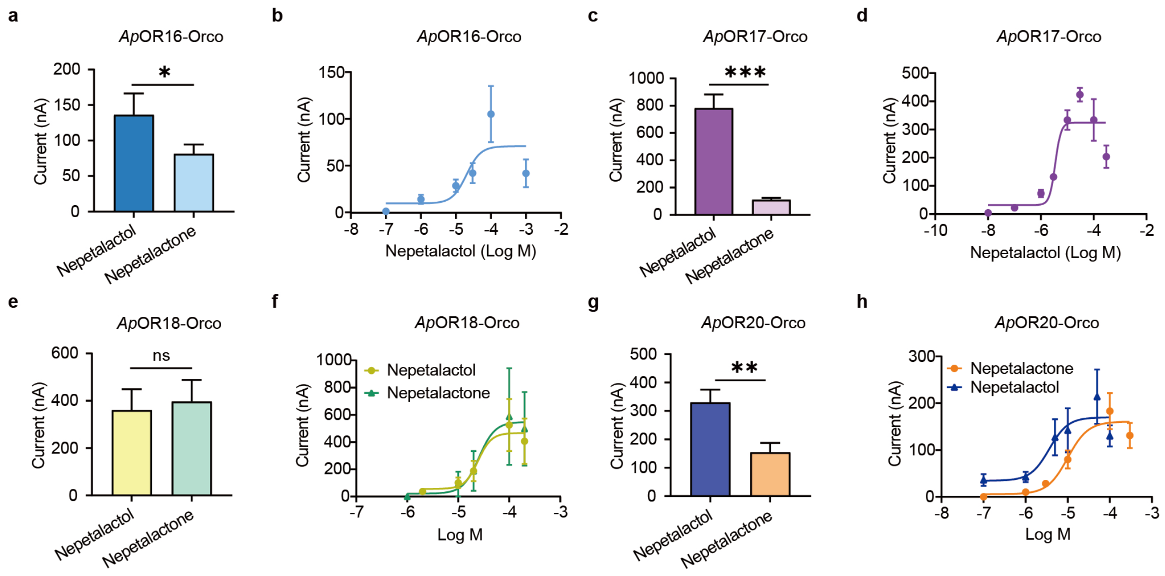

Supplementary information, Fig. S3 Functional characterization of the candidate pheromone receptors in *A. pisum*. a, c, e, g Inward current responses of *Ap*OR16-Orco (**a**, *$P < 0.05$, $n = 4$), *Ap*OR17-Orco (**c**, ***$P < 0.001$, $n = 6$), *Ap*OR18-Orco (**e**, ns, $P > 0.05$, $n = 10$), *Ap*OR20-Orco (**g**, **$P < 0.01$, $n = 6$), in response to nepetalactol and nepetalactone. **b, d, f, h** Dose-response curves. (**b**) *Ap*OR16-Orco to nepetalactol ($EC_{50} = 1.998 \times 10^{-5}$ mol/L, $n = 6$), (**d**) *Ap*OR17-Orco to nepetalactol ($EC_{50} = 3.500 \times 10^{-6}$ mol/L, $n = 3$), (**f**) *Ap*OR18-Orco to nepetalactol ($EC_{50} = 2.447 \times 10^{-5}$ mol/L, $n = 4$) and to nepetalactone ($EC_{50} = 2.537 \times 10^{-5}$ mol/L, $n = 4$), (**h**) *Ap*OR20-Orco to nepetalactol ($EC_{50} = 3.640 \times 10^{-6}$ mol/L, $n = 5$) and to nepetalactone ($EC_{50} = 9.999 \times 10^{-6}$ mol/L, $n = 5$). Data are presented as mean ± SEM.
