# Supplementary material for: Structural basis of sex pheromone detection in aphids
Source: Cell Res. 2026 Jun 22;36(8):582–94. doi: 10.1038/s41422-026-01267-z (PMC13424144; doi:10.1038/s41422-026-01267-z)
Supplement: Supplementary file 4 — Supplementary information, Fig. S4 [file 41422_2026_1267_MOESM4_ESM.pdf]

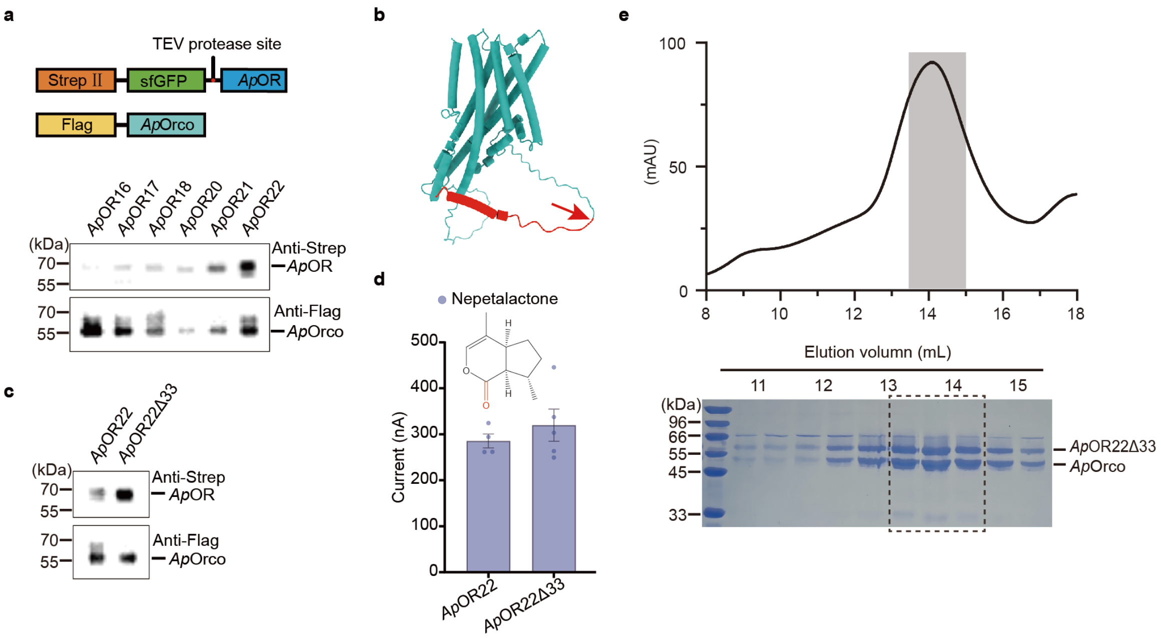

Supplementary information, Fig. S4 Protein purification of the *Ap*OR22-Orco complex. **a** Tagging schematic for *Ap*OR and *Ap*Orco with western blot confirming protein expression. **b** Schematic of N-terminal truncation in *Ap*OR22, modeled by AlphaFold2. **c** Western blot validating expression of wild-type *Ap*OR22 and truncated *Ap*OR22Δ33. **d** Electrophysiological responses of *Ap*OR22 and *Ap*OR22Δ33 to nepetalactone (Student's t-test, P = 0.439). Error bars represent SEM (n = 4-5). **e** Size-exclusion chromatography of *Ap*OR22Δ33-Orco. Peak fractions (light gray) were collected and concentrated for cryo-EM analysis.
