# Supplementary material for: Structural basis of sex pheromone detection in aphids
Source: Cell Res. 2026 Jun 22;36(8):582–94. doi: 10.1038/s41422-026-01267-z (PMC13424144; doi:10.1038/s41422-026-01267-z)
Supplement: Supplementary file 5 — Supplementary information, Fig. S5 [file 41422_2026_1267_MOESM5_ESM.pdf]

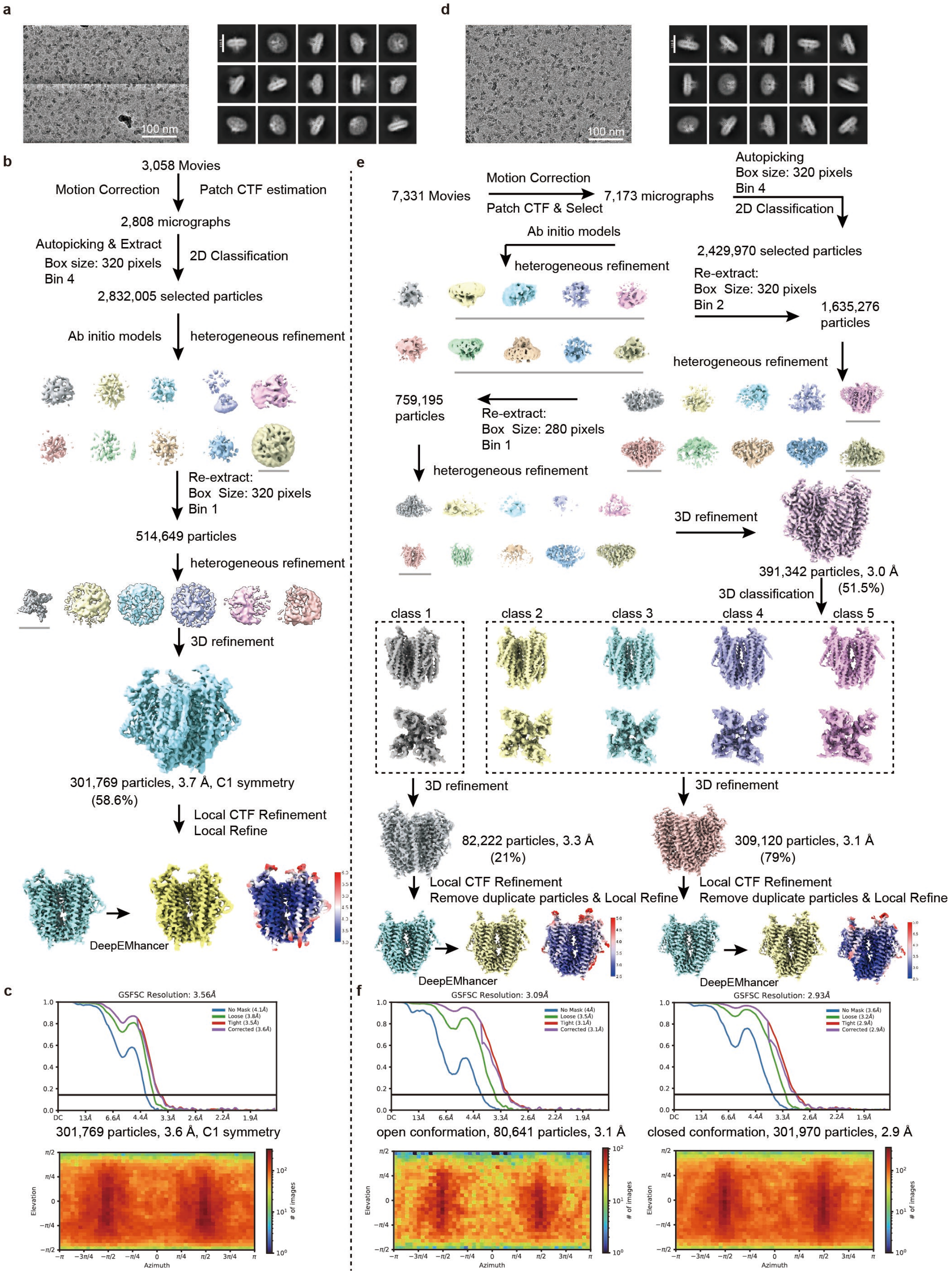

Supplementary information, Fig. S5 Cryo-EM data processing of *Ap*OR22-Orco. a, d Representative cryo-EM micrographs of *Ap*OR22-Orco in the unbound (**a**) and nepetalactone-bound (**d**) states. **b, e** Data-processing workflows and local resolution maps of final structures. **c, f** Top: gold-standard FSC (GSFSC)

curves comparing cryo-EM density maps with atomic models. Bottom: angular distribution of particles in final reconstructions. See Methods for details.
