# Supplementary material for: Structural basis of sex pheromone detection in aphids
Source: Cell Res. 2026 Jun 22;36(8):582–94. doi: 10.1038/s41422-026-01267-z (PMC13424144; doi:10.1038/s41422-026-01267-z)
Supplement: Supplementary file 6 — Supplementary information, Fig. S6 [file 41422_2026_1267_MOESM6_ESM.pdf]

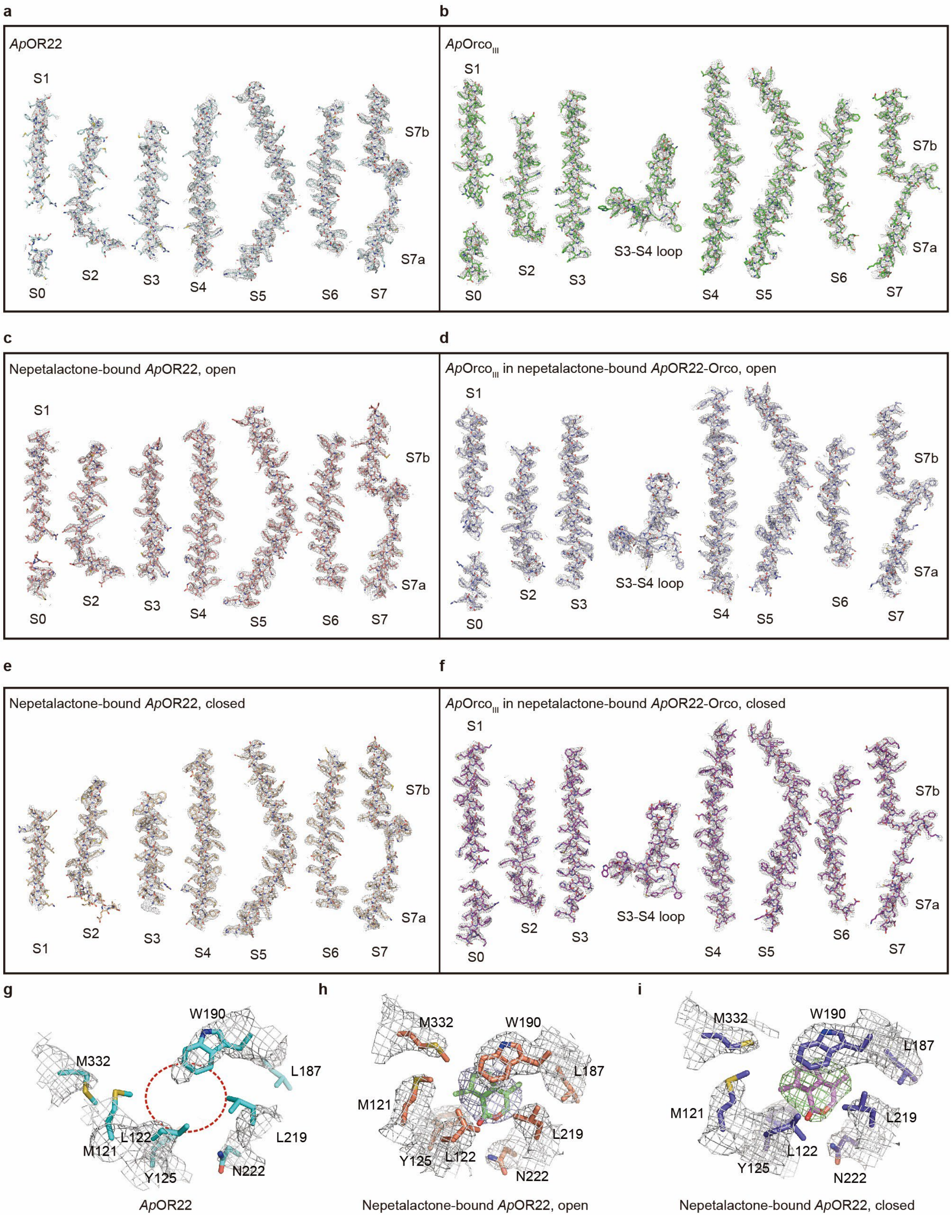

Supplementary information, Fig. S6 Cryo-EM density of *Ap*OR22-Orco. a–f Cryo-EM densities of transmembrane helices in *Ap*OR22-Orco in unbound closed (**a, b**), nepetalactone-bound open (**c, d**), and nepetalactone-bound closed (**e, f**) states. **g–i** Cryo-EM densities of the nepetalactone-binding site with or without ligand in the unbound closed (**g**), nepetalactone-bound open (**h**), and nepetalactone-bound closed (**i**) states. Red dotted circle indicates absence of ligand density in the unbound closed state (**g**).
