# Supplementary material for: Structural basis of sex pheromone detection in aphids
Source: Cell Res. 2026 Jun 22;36(8):582–94. doi: 10.1038/s41422-026-01267-z (PMC13424144; doi:10.1038/s41422-026-01267-z)
Supplement: Supplementary file 8 — Supplementary information, Fig. S8 [file 41422_2026_1267_MOESM8_ESM.pdf]

a

Rank	Docking score （kcal/mol ）
1	-6.79 ± 0.65 (182)
2	-6.62 ± 0.18 (24)
3	-5.47 (50)

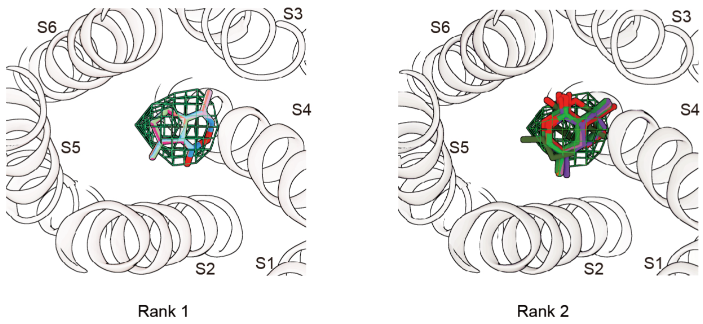

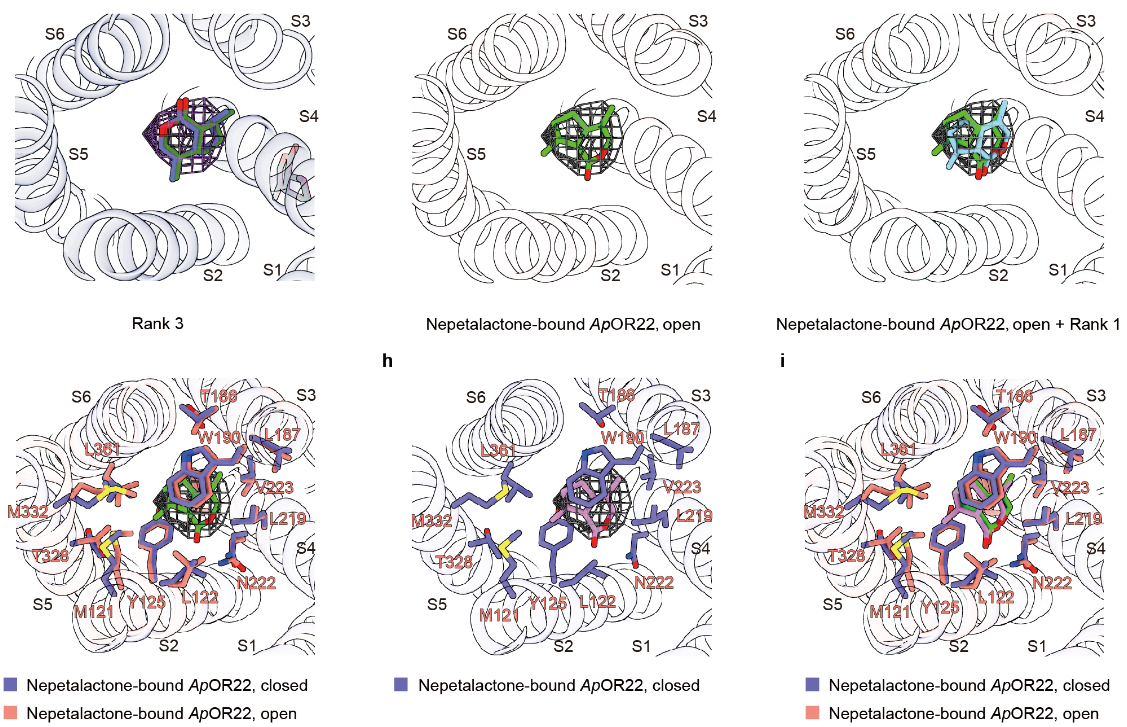

Supplementary information, Fig. S8 Ligand binding model of nepetalactone in *Ap*OR22. a Three ranked nepetalactone docking configurations with binding energy. Numbers in parentheses denote pose counts. **b–d** Superimpositions of nepetalactone docking poses from Rank 1 (9 poses), Rank 2 (7 poses), and Rank 3 (9 poses). **e** Refined nepetalactone pose in the cryo-EM ligand-bound open state. **f** Comparison of the lowest-energy Rank 1 pose with the refined cryo-EM pose. **g** Close-up of ligand-binding residues in *Ap*OR22 in nepetalactone-bound closed (purple) and open (salmon) states, with nepetalactone (lime) from the open state and density map from the closed state. **h** Refined nepetalactone pose in the cryo-EM ligand-bound closed state. **i** Superimposition of *Ap*OR22 structures in the nepetalactone-bound closed (purple) and open (salmon) states.
