# Supplementary material for: Structural basis of sex pheromone detection in aphids
Source: Cell Res. 2026 Jun 22;36(8):582–94. doi: 10.1038/s41422-026-01267-z (PMC13424144; doi:10.1038/s41422-026-01267-z)
Supplement: Supplementary file 9 — Supplementary information, Fig. S9 [file 41422_2026_1267_MOESM9_ESM.pdf]

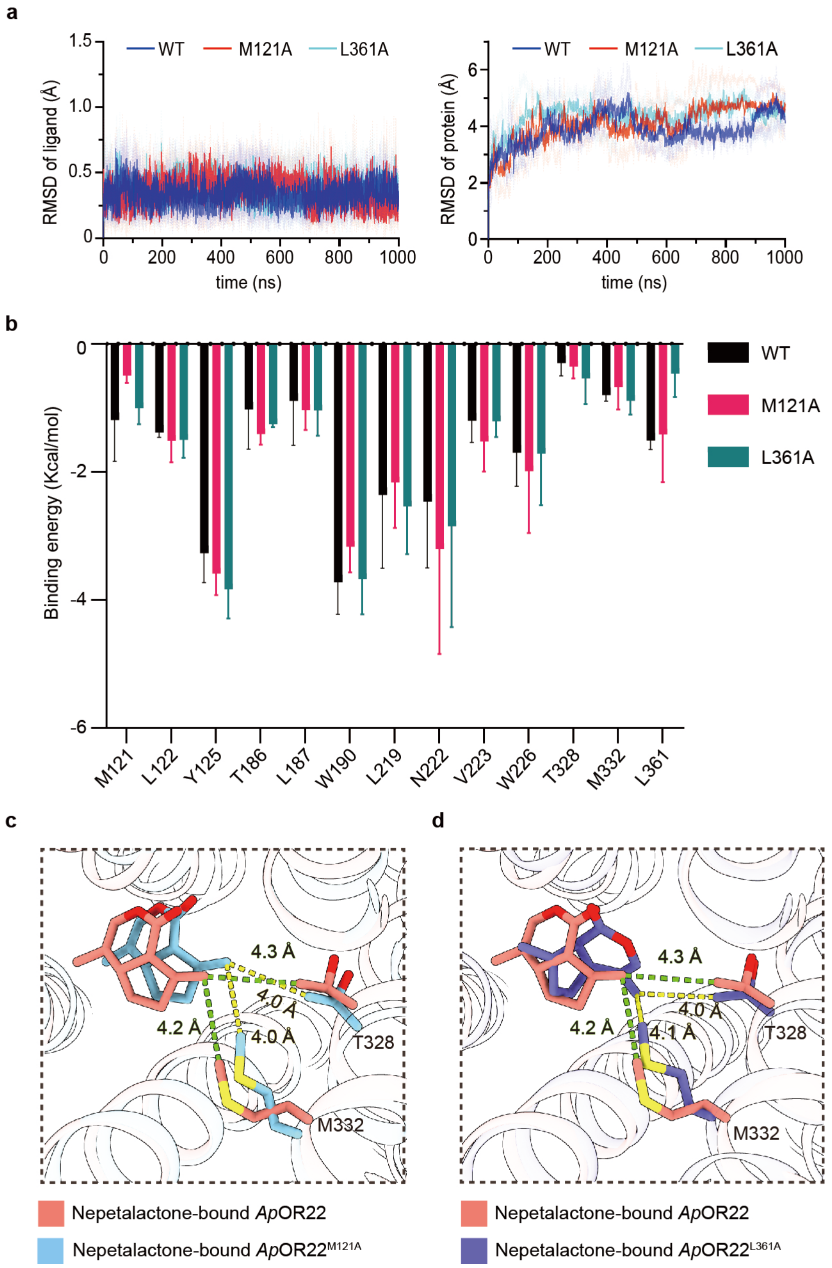

Supplementary information, Fig. S9 Molecular dynamics (MD) simulation analysis of ligand-bound *Ap*OR22 and mutants. a Average Root-Mean-Square Deviation (RMSD) values for nepetalactone (left) and the protein (wild-type *Ap*OR22, *Ap*OR22^{M121A}, *Ap*OR22^{L361A} variants; right) during triplicate 1000 ns MD simulations. **b** Ligand binding energies of nepetalactone with wild-type *Ap*OR22 and *Ap*OR22^{M121A}, *Ap*OR22^{L361A} mutants. **c, d** Structural comparison of the final MD snapshots for nepetalactone-bound *Ap*OR22 and the corresponding mutants *Ap*OR22^{M121A} (**c**) and *Ap*OR22^{L361A} (**d**). In both the mutants, residues T328 and M332 on the S5 helix shift slightly toward the ligand relative to their positions in the wild-type structure.
