# Supplementary material for: Structural basis of sex pheromone detection in aphids
Source: Cell Res. 2026 Jun 22;36(8):582–94. doi: 10.1038/s41422-026-01267-z (PMC13424144; doi:10.1038/s41422-026-01267-z)
Supplement: Supplementary file 10 — Supplementary information, Fig. S10 [file 41422_2026_1267_MOESM10_ESM.pdf]

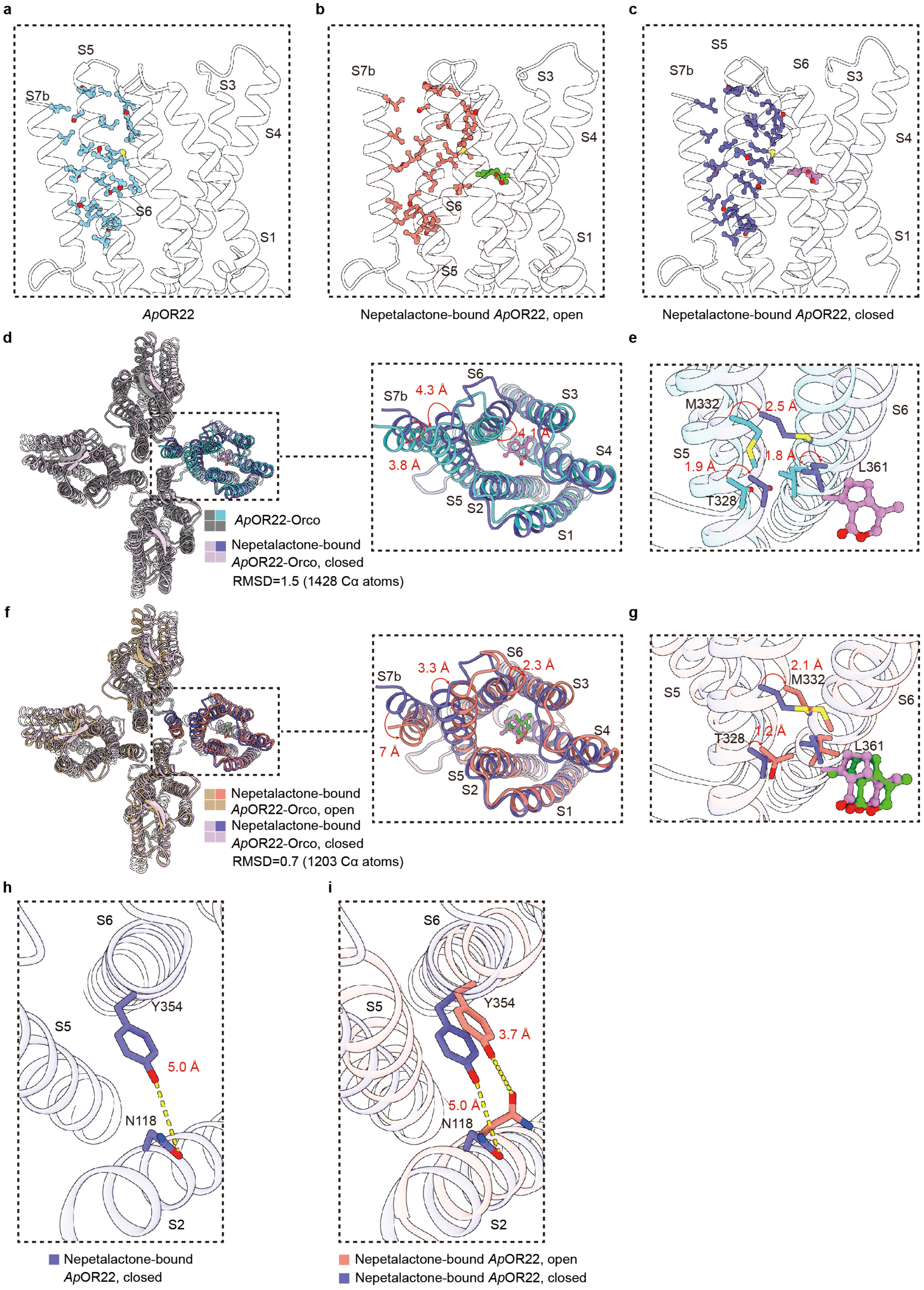
a
b
c
S5
S7b
S3
S4
S6
S1
S2
ApOR22
Nepetalactone-bound ApOR22, open
Nepetalactone-bound ApOR22, closed
d
e
4.3 Å
4.1 Å
3.8 Å
2.5 Å
1.8 Å
1.9 Å
M332
T328
L361
ApOR22-Orco
Nepetalactone-bound ApOR22-Orco, closed
RMSD=1.5 (1428 Cα atoms)
f
g
3.3 Å
2.3 Å
7 Å
2.1 Å
1.2 Å
Nepetalactone-bound ApOR22-Orco, open
Nepetalactone-bound ApOR22-Orco, closed
RMSD=0.7 (1203 Cα atoms)
h
i
Y354
N118
5.0 Å
3.7 Å
Nepetalactone-bound ApOR22, closed
Nepetalactone-bound ApOR22, open

Supplementary information, Fig. S10 Conformational changes of *Ap*OR22-Orco upon ligand binding
a–c Close-up views of interacting residues on S5, S6, and S7b helices within 5 Å of each other in unbound closed (**a**), nepetalactone-bound open (**b**), and nepetalactone-bound closed (**c**) states. Residues are shown as ball-sticks. **d, f** Superimpositions of the nepetalactone-bound closed structure with the unbound closed (**d**) and nepetalactone-bound open (**f**) states, viewed extracellularly. Expanded views highlight helix rotations (red arrows). **e, g** Close-ups of residue differences between nepetalactone-bound closed and unbound closed states (**e**), and between nepetalactone-bound closed and nepetalactone-bound open states (**g**). Red arrows denote residue rotations. Nepetalactone is depicted as pink ball-sticks (nepetalactone-bound closed) or lime ball-sticks (nepetalactone-bound open). **h, i** A hydrogen bond between N118 and Y354 forms in the nepetalactone-bound open state but is absent in the nepetalactone-bound closed state. Yellow dashed lines indicate the N118-Y354 distance.
