# Supplementary material for: Structural basis of sex pheromone detection in aphids
Source: Cell Res. 2026 Jun 22;36(8):582–94. doi: 10.1038/s41422-026-01267-z (PMC13424144; doi:10.1038/s41422-026-01267-z)
Supplement: Supplementary file 11 — Supplementary information, Table. S1 [file 41422_2026_1267_MOESM11_ESM.pdf]

Table. S1 Interaction affinities between 87 odorant receptors in *A. pisum* and nepetalactol/nepetalactone.

*Ap*OR	Delta (Kcal/mol)		*Ap*OR	Delta (Kcal/mol)		*Ap*OR	Delta (Kcal/mol)	
	Nepetalactol	Nepetalactone		Nepetalactol	Nepetalactone		Nepetalactol	Nepetalactone
OR2	-25	#	OR32	-27		OR61*	-27	-20
OR3	-31	-28	OR33	-27	-30	OR62*	\	\
OR4	-32	-30	OR34	-30	-32	OR64	-29	-27
OR5	\	-26	OR35	-31	-25	OR65	-29	-24
OR6	-26	-26	OR36	-32	-29	OR66	-28	-25
OR7	-29	-29	OR37	-32	-27	OR67	-31	43
OR8	-30	-27	OR38	-35	-27	OR68	-23#	-22#
OR9	-24	-28	OR39	-32	-34	OR69	-25	-24
OR10	-28	-24	OR40	-30	-29	OR70	-24	-26
OR11	-26	-24	OR41	52	-23	OR71	#	#
OR12	-25	\	OR42	-27	-20	OR72	-25	-23
OR13	-31	-29	OR43	-26	302	OR73*	\	\
OR14	-29	-25	OR44	-24	-25	OR74	6	-25
OR15	-22	-20#	OR45	-28	-27	OR75	-26	-27
OR16	-31	-29	OR46	-25	-22	OR76	-24	-17
OR17	-31	-29	OR47	-29	-26	OR77	-23	-25
OR18	-31	-27	OR48	-30	-28	OR78	-29	275
OR20	-33	-31	OR49	-31	-33	OR79	-33	-31
OR21	-32	-25	OR50	-29	-23	OR80	-30	\
OR22	-31	-35	OR51	-27	65	OR81	-36	-34
OR23	-32	-27	OR52	-23	-21	OR82	-29	99
OR24	-29	-26	OR53	-30	-24	OR83	-23	-27
OR25	-30	-25	OR54	769	-29	OR84	-28	-22
OR26	-26	-25	OR55	\	-26#	OR85	-19#	-15#
OR27	\	\	OR56	-26	-29	OR86	-20	-24
OR28	490	-24	OR57*	\	\	OR87	-25	-11
OR29	-27	-25	OR58*	\	\	OR88	-29	-27
OR30	-30	-27	OR59*	\	\	OR89	\	-25
OR31	-30	-27	OR60*	\	\	OR90	-28	-30

Values below -31 Kcal/mol are identified in yellow. The binding affinity was predicted using the MM/GBSA (Molecular Mechanics/Generalized Born Surface Area) approach. The asterisk (*) indicates that the predicted OR lacks an integrated conformation, while the hash (#) suggests that the ligand is not localized to the general OR pocket.
