# Supplementary material for: Structural basis of sex pheromone detection in aphids
Source: Cell Res. 2026 Jun 22;36(8):582–94. doi: 10.1038/s41422-026-01267-z (PMC13424144; doi:10.1038/s41422-026-01267-z)
Supplement: Supplementary file 12 — Supplementary information, Table. S2 [file 41422_2026_1267_MOESM12_ESM.pdf]

Table S2. Chemicals used for TEVC screening

Number	Chemicals	CAS number
1	6-Methyl-5-hepten-2-one	110-93-0
2	(S)-(-)-Limonene	5989-54-8
3	(-)-β-Pinene	18172-67-3
4	α-Pinene	80-56-8
5	Geranyl acetate	105-87-3
6	(*E*)-β-Farnesene	18794-84-8
7	(*E*, *E*)-Trimethyl-1,3,7,12-tridecatetraene	62235-06-7
8	(-)-Myrtenal	18486-69-6
9	β-Ionone	14901-07-6
10	(-)-α-Cedrene	469-61-4
11	Sabinene	3387-41-5
12	(-)-Verbenone	1196-01-6
13	Isolongifolene	1135-66-6
14	Camphene	79-92-5
15	(S)-*cis*-Verbenol	18881-04-4
16	Cumene	98-82-8
17	β-Caryophyllene	87-44-5
18	Farnesol	4602-84-0
19	6,10-Dimethyl-5,9-undecadien-2-one	689-67-8
20	Geraniol	106-24-1
21	Methyl salicylate	119-36-8
22	Methyl eugenol	93-15-2
23	Eugenol	97-53-0
24	2-Phenylethanol	60-12-8
25	Benzaldehyde	100-52-7
26	Cinnamaldehyde	104-55-2
27	Benzyl alcohol	100-51-6
28	Phenylacetaldehyde	122-78-1
29	Methyl 2-methoxybenzoate	606-45-1
30	Hexanal	66-25-1
31	*cis*-2-Hexen-1-ol	928-94-9
32	*trans*-2-Hexen-1-ol	928-95-0
33	2,3-Butanediol	513-85-9
34	*cis*-3-Hexenyl Acetate	3681-71-8
35	*cis*-3-Hexen-1-ol	928-96-1
36	3-Methyl-2-butenal	107-86-8
37	*trans*-2-Hexenal	6728-26-3
38	(-)-Borneol	464-45-9
39	Citral	5392-40-5
40	Butyraldehyde	123-72-8

41	2,3-Butanedione	431-03-8
42	Nerolidol	7212-44-4
43	1-Hexanol	111-27-3
44	(-)-Myrtenol	19894-97-4
45	Acetoin	513-86-0
46	Indole	120-72-9
47	Methyl jasmonate	39924-52-2
48	Jasmone	488-10-8
49	Citronellol	106-22-9
50	*p*-Cresol	106-44-5
51	Phenol	108-95-2
52	1-Octen-3-ol	3391-86-4
53	α-Ionone	127-41-3
54	Vanillin	121-33-5
55	(+)-Borneol	464-43-7
56	Cedrol	77-53-2
57	2-Heptanone	110-43-0
58	Eucalyptol	470-82-6
59	(+)-(4*aS*,7*S*,7*aR*)-Nepetalactone	109215-55-6
60	(-)-(1*R*,4*aS*,7*S*,7*aR*)-Nepetalactol	21651-62-7
