# Supplementary material for: Structural basis of sex pheromone detection in aphids
Source: Cell Res. 2026 Jun 22;36(8):582–94. doi: 10.1038/s41422-026-01267-z (PMC13424144; doi:10.1038/s41422-026-01267-z)
Supplement: Supplementary file 13 — Supplementary information, Table. S3 [file 41422_2026_1267_MOESM13_ESM.pdf]

Table. S3 Cryo-EM data collection and refinement statistics

	*Ap*OR22-Orco heterocomplex (PDB ID, 9WPF; EMDB ID, EMD-66141)	*Ap*OR22-Orco heterocomplex bound with nepetalactone in the closed state. (PDB ID, 9WPE; EMDB ID, EMD-66140)	*Ap*OR22-Orco heterocomplex bound with nepetalactone in the open state. (PDB ID, 9WPG; EMDB ID, EMD-66142)
**Data collection and Processing**			
Microscope	Krios	Krios	
Voltage(keV)	300	300	
Camera	Gatan K3	Gatan K3	
Magnification	105,000	105,000	
Pixel size at detector (Å/pixel)	0.82	0.824	
Total electron exposure (e–/Å2)	50	50	
Exposure time (s)	1.9	2.16	
Number of frames collected during exposure	40	40	
Defocus range (μm)	-1.0 ~ -2.0	-1.3 ~ -1.8	
Automation software	EPU v2.9	EPU v2.9	
Energy filter slit width	20 eV	20 eV	
Micrographs collected (no.)	3,058	7,331	
Micrographs used (no.)	2,808	7,173	
Total extracted particles (no.)	3,048,328	2,624,051	
**For each reconstruction:**			
Final particles (no.)	301,769	301,970	80.641
Point-group	C1	C1	C1
Resolution (global, Å)	3.6	2.9	3.1
FSC 0.5 (unmasked/masked)	4.5/3.9	4.2/3.3	6.9/3.5
FSC 0.143 (unmasked/masked)	4.1/3.5	3.6/2.9	4.0/3.1
Resolution range (local, Å)	3.0-6.0	2.5-5.0	2.5-5.0
Map sharpening *B* factor (Å2)	160.6	108.3	92.3
Map sharpening methods	Half-maps correlation	Half-maps correlation	Half-maps correlation
**Model composition**			
Protein	1,560	1,536	1,522
Ligands/Nucleotide	6	7	4
**Model Refinement**			
Refinement package	PHENIX	PHENIX	PHENIX
- real or reciprocal space	Real space	Real space	Real space

- resolution cutoff	3.6	2.9	3.1
Model-Map scores			
- CCvolume/CCmask	0.72/0.71	0.79/0.79	0.78/0.78
B factors (Å$_2$)			
-Protein residues	88.90	69.97	72.16
-Ligand	85.41	90.88	76.58
R.m.s. deviations from ideal values			
-Bond lengths (Å)	0.005	0.005	0.006
-Bond angles (°)	0.580	0.541	0.551
**Validation**			
MolProbity score	1.98	1.33	1.25
CaBLAM outliers	2.56	2.13	0.56
Clashscore	5.51	3.96	2.63
Poor rotamers (%)	2.91	0.86	1.53
C-beta deviations	0	0	0
**Ramachandran plot**			
Favored (%)	95.33	97.17	97.73
Outliers (%)	0	0	0.07

Cryo-EM data collection, refinement, and validation statistics for the *Ap*OR22-Orco complex in the unbound, nepetalactone-bound closed, and nepetalactone-bound open states.
