# Supplementary material for: Structural basis of sex pheromone detection in aphids
Source: Cell Res. 2026 Jun 22;36(8):582–94. doi: 10.1038/s41422-026-01267-z (PMC13424144; doi:10.1038/s41422-026-01267-z)
Supplement: Supplementary file 14 — Supplementary information, Table. S4 [file 41422_2026_1267_MOESM14_ESM.pdf]

Table S4. Primers used in RT-qPCR

Primer name	Sequence (5’-3’)
Primers for dsRNA synthesis	
*Ap*OR21-F-T7	**TAATACGACTCACTATAGGGAGA**CGATTACGATGACCAGCCTT
*Ap*OR21-R-T7	**TAATACGACTCACTATAGGGAGA**AAATCGTCATAATGGCTCGG
*Ap*OR22-F-T7	**TAATACGACTCACTATAGGGAGA**GGCGGAACGGCCATGGA
*Ap*OR22-R-T7	**TAATACGACTCACTATAGGGAGA**GTGAGTATGATAGACTCGACGACGTAC
dsGFP-F-T7	**TAATACGACTCACTATAGGGAGA**CTGGACGGCGACGTAAACG
dsGFP-R-T7	**TAATACGACTCACTATAGGGAGA**CCTTCGGGCATGGCGGAC
Primers for RT-qPCR	
*Ap*OR21-qF	GCTTCGCTCACTATTGAACTGTAC
*Ap*OR21-qR	CATCACTCGCTTGTGAGCAGAG
*Ap*OR22-qF	ACACCTACGGTTCTGCTTCAAA
*Ap*OR22-qR	AATCCTTGTCCGTCCAATCACA
*Ap*Tubulin-qF	TTGGTACACTGGTGAAGGTATG
*Ap*Tubulin-qR	AGCGGTAGCTTCTTGGTATTG
*Ap*Actin-qF	CGTTACCAACTGGGACGATATG
*Ap*Actin-qR	GGGTTCAATGGAGCTTCTGTTA
