# Supplementary material for: Structural basis of sex pheromone detection in aphids
Source: Cell Res. 2026 Jun 22;36(8):582–94. doi: 10.1038/s41422-026-01267-z (PMC13424144; doi:10.1038/s41422-026-01267-z)
Supplement: Supplementary file 15 — Supplementary information, Data. S1 [file 41422_2026_1267_MOESM15_ESM.pdf]

>AcraORco
MGYKKEGLIKDLWPNIRLIQLSGLFISEYYDDYSGLAVLLRKIYSWITTIIIYSQF
IFIVIFMVTKSNDSDQLAAGVVTTLFFTHSMIKFMYFSTGTKSFYRTLSCWNN
TSPHPLFAESHSRFHAKSLSRMRQLLIIVSIVTIFTTISWTTITFFGESVWKVPDP
ESFNQTMYIPVPRLMLHSWYPWDSSNGLGYIVAFILQFYWIFITLSHSNLLELL
FSSFLVHACEQLQHLKEILNPLIELSATLDSAVRNPAEIFRANSAKNQPINGVGK
LLFIKSFIYHFGMS
>AfabORco
MGYKKDGLIKDLWPNIRLIQLSGLFISEYYDDYSGLAVLLRKIYSWITTIIIYSQ
FIFIVIFMVTKSNDSDQLAAGVVTTLFFTHSMIKFMYFSTGTKSFYRTLSCWNN
TSPHPLFTESHSRFHAKSLSRMRQLLIIVSIVTIFTTISWTTITFFGESVWKVPDP
ETFNQTMYIPVPRLMLHSWYPWDSSYGLGYIVAFVLQFYWIFITLSHSNLLEL
LFSSFLVHACEQLQHLKEILNPLIELSATLDSAVRNPAEIFRANSAKNQPINGVEI
DYNGSYVNEITEYGTKGETELNRKGPNNLTSNQEVLVRSAIKYWVERHKHVV
KYVSLITECYGSALLFHMLVSTVILTILAYQATKINGVNVFAFSTIGYLMYSFAQ
IFMFCIHGNELIEESSSVMEAAYGCQWYDGSEEAKTFVQIVCQQCQKPLIVSG
AKFFNVSLDLFASVLGAVVTYFMVLVQLK
>AgosORco
MGYKKDGLIKDLWPNIRLIQLSGLFISEYYDDYSGLAVLLRKIYSWITTIIIYSQ
FIFIVIFMVTKSNDSDQLAAGVVTTLFFTHSMIKFMYFSTGTKSFYRTLSCWNN
TSPHPLFTESHSRFHAKSLSRMRQLLIIVSIVTIFTTISWTTITFFGESVWKVPDP
ETFNQTMYIPVPRLMLHSWYPWDSSHGLGYIVAFALQFYWIFITLSHSNLLEL
LFSSFLVHACEQLQHLKEILNPLIELSATLDSAVHNPAEIFRANSAKNQPINGVEI
DYNGSYVNEITEYGTKGETELNRKGPNNLTSNQEVLVRSAIKYWVERHKHVV
KYVSLITECYGSALLFHMLVSTVILTILAYQATKINGVNVFAFSTIGYLMYSFAQ
IFMFCIHGNELIEESSSVMEAAYGCQWYDGSEEAKTFVQIVCQQCQKPLIVSG
AKFFNVSLDLFASVLGAVVTYFMVLVQLK
>ArumORco
MGYKKDGLIKDLWPNIRLIQLSGLFISEYYDDYSGLAVLLRKIYSWTTTIIIYSQ
FIFIVIFMVTKSNDSDQLAAGVVTTLFFTHSMIKFMYFSTGTKSFYRTLSCWNN
TSPHPLFTESHSRFHAKSLSRMRQLLIIVSIVTIFTTISWTTITFFGESVWKVPDP
ETFNQTMYIPVPRLMLHSWYPWDSSYGLGYIVAFVLQFYWIFITLSHSNLLEL
LFSSFLVYACEQLQHLKEILNPLIELSATLDSAVRNPAEIFRANSAKNQPINGVEI
DYNGSYVNEITEYGTKGEAELNRKGPNNLTSNQEVLVRSAIKYWVERHKHV
VKYVSLITECYGSALLFHMLVSTVILTILAYQATKINGVNVFAFSTIGYLMYSFA
QIFMFCIHGNELIEESSSVMEAAYGCQWYDGSEEAKTFVQIVCQQCQKPLIVS
GAKFFNVSLDLFASVLGAVVTYFMVLVQLK
>AthaORco
MGYKKDGLIKDLWPNIRLIQLSGLFISEYYDDYSGLAMLLRKIYSWITTIIIYSQ
FIFIVIFMVTKSNDSDQLAAGVVTTLFFTHSMIKFMYFSTGTKSFYRTLSCWNN
TSPHPLFTESHSRFHAKSLSRMRQLLIIVSIVTIFTTISWTTITFFGESVWKVPDP
ETFNQTMYIPVPRLMLHSWYPWDSSHGLGYIVAFVLQFYWIFITLSHSNLLEL
LFSSFLVHACEQLQHLKEILNPLIELSATLDSAVHNPAEIFRANSAKNQPINGVEI
DYNGSYVNEITEYGTKGETELNRKGPNKLTSNQEVLVRSAIKYWVERHKHVV

KYVSLITECYGSALLFHMLVSTVILTILAYQATKINGVNVFAFSTIGYLLYSFAQI
FMFCIHGNELIEESSSVMEAAYSCQWYDGSEEAKTFVQIVCQQCQKPLIVSGA
KFFNVSLDLFASVLGAVVTYFMVLVQLK
>MvarORco
MGYKKDGLIKDLWPNIRLIQMSGLFISEYYDDYSGLAVLFRKIYSWITAIIIYSQ
FIFIVMFMVTKSNDSDQLAAGVVTTLFFTHSMIKFMYFSTGTKSFYRTLSCWN
NTSPHPLFTESHSRFHAKSLSRMRQLLIIVSIVTIFTTISWTTITFFGESVWKVPN
PETFNQTMYVPVPRLMLHSWYPWDASRGLGYIVAFVLQFYWIFITLSHSNLM
ELLFSSFLVHACEQLQHLKEILNPLIELSATLDSSVHNPAEIFRASSAKNQSINGI
DHDYNGSYVNEITEYGTKGENELNRKGPNNLTSNQEVLVRSAIKYWVERHK
HIVKYVSLITECYGSALLFHMLVSTVILTILAYQATKINGVNVFAFSTIGYLMYS
FAQIFMFCIHGNELIEESSSVMEAAYGCHWYDGSEEAKTFVQIVCQQCQKPLI
VSGAKFFNVSLDLFASVLGAVVTYFMVLVQLK
>McerORco
MGYKKDGLIKDLWPNIRLIQMSGLFISEYYDDYSGLAVLFRKIYSWITTIIIYSQ
YIFIVIFMVTKSNDSDQLAAGVVTTLFFTHSMIKFMYFSTGTKSFYRTLSCWN
NTSPHPLFTESHSRFHARSLSRMRQLLIIVSIVTIFTTISWTTITFFGESVWKVPN
PETFNQTMYVPVPRLMLHSWYPWDASRGLGYIVAFVLQFYWIFITLSHSNLM
ELLFSSFLVHACEQLQHLKEILNPLIELSATLDSSVHNPAEIFRANSAKNQSINGI
DRDYNGSYVNEITEYGTKGENESNRKGPNNLTSNQEVLVRSAIKYWVERHKH
VVKYVSLITDCYGSALLFHMLVSTVILTILAYQATKINGVNVFAFSTIGYLLYSF
AQIFMFCIHGNELIEESSSVMEAAYGCHWYDGSEEAKTFVQIVCQQCQKPLIV
SGAKFFNVSLDLFASVLGAVVTYFMVLVQLK
>MlytORco
MGYKKDGLIKDLWPNIRLIQMSGLFISEYYDDYSGLAVLFRKIYSWITTIIIYSQ
FIFIVIFMVTKSNDSDQLAAGVVTTLFFTHSMIKFMYFSTGTKSFYRTLSCWNN
TSPHPLFTESHSRFHAKSLSRMRQLLIIVSIVTIFTTISWTTITFFGESVWKVPNP
ETFNQTMYVPVPRLMLHSWYPWDASRGLGYIVAFVLQFYWIFITLSHSNLME
LLFSSFLVHACEQLQHLKEILNPLIELSATLDSSVHNPAEIFRANSAKNQSINGID
RDYNGSYVNEITEYGTKGENEPNRKGPNNLTSNQEVLVRSAIKYWVERHKHV
VKYVSLITDCYGSALLFHMLVSTVILTILAYQATKINGVNVFAFSTIGYLLYSFA
QIFMFCIHGNELIEESSSVMEAAYGCHWYDGSEEAKTFVQIVCQQCQKPLIVS
GAKFFNVSLDLFASVLGAVVTYFMVLVQLK
>RmaiORco
MGYKKDGLIKDLWPNIRLIQMSGLFISEYYDDYSGLAVLFRKIYSWITTIIIYSQ
FIFIVIFMVTKSNDSDQLAAGVVTTLFFTHSMIKFMYFSTGTKSFYRTLSCWNN
TSPHPLFTESHSRFHAKSLSRMRQLLIIVSIVTIFTTISWTTITFFGESVWKVPNP
ETFNQTMYIPVPRLMLHSWYPWDASRGLGYIVAFVLQFYWIFITLSHSNLLEL
LFSSFLVHACEQLQHLKEILNPLIELSATLDSAVHNPAEIFRANSAKNQPINGVEI
DYNGSYVNEITEYGTKGETEPNRKGPNNLTSNQEVLVRSAIKYWVERHKHVV
KYVSLITECYGSALLFHMLVSTVILTILAYQATKINGVNVFAFSTIGYLLYSFAQI
FMFCIHGNELIEESSSVMEAAYGCQWYDGSEEAKTFVQIVCQQCQKPLIVSGA
KFFNVSLDLFASVLGAVVTYFMVLVQLK

>RpadORco
MGYKKDGLIKDLWPNIRLIQMSGLFISEYYDDYSGLAVLFRKIYSWITTIIIYSQ
FIFIVIFMVTKSNDSDQLAAGVVTTLFFTHSMIKFMYFSTGTKSFYRTLSCWNN
TSPHPLFTESHSRFHAKSLSRMRQLLIIVSIVTIFTTISWTTITFFGESVWKVPNP
ETFNQTMYIPVPRLMLHSWYPWDASRGLGYIVAFVLQFYWIFITLSHSNLLEL
LFSSFLVHACEQLQHLKEILNPLIELSATLDSAVHNPAEIFRANSAKNQPINGVEI
DYNGSYVNEITEYGTKGDTEPNRKGPNNLTSNQEVLVRSAIKYWVERHKHVV
KYVSLITECYGSALLFHMLVSTVILTILAYQATKINGVNVFAFSTIGYLMYSFAQ
IFMFCIHGNELIEESSSVMEAAYGCQWYDGSEEAKTFVQIVCQQCQKPLIVSG
AKFFNVSLDLFASVLGAVVTYFMVLVQLK
>SaveORco
MYTFSTNMGYKKDGLIKDLWPNIRLIQMSGLFISEYYEDYSGLAVLFRKIYSW
ITTIIIYSQFIFIVMFMVTKSYDSDQLAAGVVTTLFFTHSMIKFMYFSTGTKSFY
RTLSCWNNTSPHPLFAESHSRFHAKSLSRMRQLLIIVSIVTIFTTISWTTITFFGE
SVWKVPNPETFNQTMYVPVPRLMLHSWYPWDSSHGLGYIVAFVLQFYWIFIT
LSHSNLMELLFSSFLVHACEQLQHLKEILNPLIELSATLDSSVHNPAEIFRASSA
KNQSINGIDRDYNGSFVNEITEYGTKGENETNRKGPNNLTSNQEVLVRSAIKY
WVERHKHVVKYVSLITECYGSALLFHMLVSTVILTILAYQATKINGVNVFAFS
TIGYLMYSFAQIFMFCIHGNELIEESSSVMEAAYGCHWYDGSEEAKTFVQIVC
QQCQKPLIVSGAKFFNVSLDLFASVLGAVVTYFMVLVQLK
>SmisORco
MYTFSTNMGYKKDGLIKDLWPNIRLIQMSGLFISEYYEDYSGLAVLFRKIYSW
ITTIIIYSQFIFIVMFMVTKSYDSDQLAAGVVTTLFFTHSMIKFMYFSTGTKSFY
RTLSCWNNTSPHPLFAESHSRFHAKSLSRMRQLLIIVSIVTIFTTISWTTITFFGE
SVWKVPNPETFNQTMYVPVPRLMLHSWYPWDSSHGLGYIVAFVLQFYWIFIT
LSHSNLMELLFSSFLVHACEQLQHLKEILNPLIELSATLDSSVHNPAEIFRASSA
KNQSINGIDRDYNGSFVNEITEYGTKGENETNRKGPNNLTSNQEVLVRSAIKY
WVERHKHVVKYVSLITECYGSALLFHMLVSTVILTILAYQATKINGVNVFAFS
TIGYLMYSFAQIFMFCIHGNELIEESSSVMEAAYGCHWYDGSEEAKTFVQIVC
QQCQKPLIVSGAKFFNVSLDLFASVLGAVVTYFMVLVQLK
>ApisORco
MYTFSTNMGYKKDGLIKDLWPNIRLIQLSGLFISEYYDDYSGLAVLFRKIYSWI
TAIIIYSQFIFIVIFMVTKSNDSDQLAAGVVTTLFFTHSMIKFVYFSTGTKSFYRT
LSCWNNTSPHPLFAESHSRFHAKSLSRMRQLLIIVSIVTIFTTISWTTITFFGESV
WKVPDPETFNQTMYVPVPRLMLHSWYPWDSSHGLGYIVAFVLQFYWIFITLS
HSNLMELLFSSFLVHACEQLQHLKEILNPLIELSATLDSSVHNPAEIFRANSAKN
QSINGIDHDYNGSYVNEITEYGTKGENEPNRKGPNNLTSNQEVLVRSAIKYWV
ERHKHVVKYVSLITECYGSALLFHMLVSTVILTILAYQATKINGVNVFAFSTIG
YLMYSFAQIFMFCIHGNELIEESSSVMEAAYGCHWYDGSEEAKTFVQIVCQQC
QKPLIVSGAKFFNVSLDLFASVLGAVVTYFMVLVQLK
>AglyORco
MGYKKDGLIKDLWPNIRLIQLSGLFISEYYDDYSGLAVLLRKIYSWITTIIIYSQ
FIFIVIFMVTKSNDSDQLAAGVVTTLFFTHSMIKFMYFSTGTKSFYRTLSCWNN
TSPHPLFTESHSRFHAKSLSRMRQLLIIVSIVTIFTTISWTTITFFGESVWKVPDP

ETFNQTMYIPVPRLMLHSWYPWDSSHGLGYIVAFALQFYWIFITLSHSNLLEL
LFSSFLVHACEQLQHLKEILNPLIELSATLDSAVHNPAEIFRANSAKNQPINGVD
YNGSYVNEITEYGTKGETELNRKGPNNLTSNQEVLVRSAIKYWVERHKHVVK
YVSLITECYGSALLFHMLVSTVILTILAYQATKINGVNVFAFSTIGYLMYSFAQI
FMFCIHGNELIEESSSVMEAAYGCQWYDGSEEAKTFVQIVCQQCQKPLIVSGA
KFFNVSLDLFASVLGAVVTYFMVLVQLK
>MperORco
MQCQPLFTRRFVLRLQILNLRNLHNIIQRIGHMAMGYKKDGLIKDLWPNIRLI
QLSGLFISEYYDDYSGLAVLLRKIYSWITTIIIYSQFIFIVMFMVTKSNDSDQLA
AGVVTTLFFTHSMIKFMYFSTGTKSFYRTLSCWNNTSPHPLFTESHSRFHAKS
LSRMRQLLIIVSIVTIFTTISWTTITFFGESVWKVPNPETFNQTMYVPVPRLMLH
SWYPWDASHGLGYIVAFVLQFYWIFITLSHSNLMELLFSSFLVHACEQLQHLK
EILNPLIELSATLDSSVHNPAEIFRATSAKNQAINGIDRDYNGSYVNEITEYGTK
GENESNRKGPNNLTSNQEVLVRSAIKYWVERHKHVVKYVSLITDCYGSALLF
HMLVSTVILTILAYQATKINGVNVFAFSTIGYLMYSFAQIFMFCIHGNELIEESSS
VMEAAYGCHWYDGSEEAKTFVQIVCQQCQKPLIVSGAKFFNVSLDLFASVLG
AVVTYFMVLVQLK
>AgosOR21
MYQLLRPVECGLDVGRCRSAALAVVFMTLGLQSMQVARLYLARHDFQMFAN
MGVLVVNGLMCLLKGYMVVANADRMCSTLDATRYAFTGCGGRDPSVLRRC
RATLSTILRTFVALSFGVWAVVYVVESTILTVNVFCWTSFDCMDQWYTKHKL
RLFRTMSTGYETLGRSRSGDVKLSARQQFDTVAAGAAISKTSITDDNLDDLKS
HIIDNKNIIEQYDAFFDVVRPMVLIQIADGSYSIITLIFLTSLVYLKGYSIVSAPIL
KFVCGLASLTIELYIYCYGFNHIEDGKSTVNFGLYSSNWTEMGF
>MperOR21
MENPERVDYDSNGVTGNPAEKAEGGEMDNPPKNDTEGGTILDVELFKIIGVY
QLLRPDEFGLNARLCRTTAIVVVCLTLGLQSMQVCRLYLARHDLQMFANVGV
MIINGLMCLLKGYMVAANADRMSATLNAARYAFTGCGNRDQSKLRLCRARL
STILRTFVRLSFGTLIVWVVMPWFMASEYDDTPSIWATVYVIESIILTVNVFCW
TSFDCYLVTMCFVLEALFCTMSTGYETLGRHRAAAKSSAGQQLAIAGTISDVS
ISDVNYDDLTSHILDNQNIIEQYDEFFDVVRPMVLVQIANGMYSIITLIFLTLLT
YLSGYSIVSAPFLKFVCGLASLTIELYIYCYGFNHIEDGKSTVNFGLYSSNWTE
MDLKFKNTLLMAMIMNSAHKRVMKVSPNSIVNLEMFTGVMNMSYSIVSVLL
N
>MvarOR21
MGNPEGVDYDSNGVKGNSAEKAEGGAMVNPAKNDTEDGTILDVELFKFIGV
YQLLRPTEFGLNAGLCRTTVIVVVCLTLGLQSMQVCRLYLARHDLQMFANVG
VMIINGLMCLLKGYMVAANADRMSATLNSARYAFTGCGNRDQSKLRLCRAR
LSTILRTFVQLSFGTLIVWVVMPWFMASEYDDTPSIWATVYVIESIILTVNVFC
WTSFDCYLVTMCFVLEALFCTMSTGYETLGRRRTDAKSSAGQQLGIAGTISD
VSISDVNYDDLKSHILDNQNIIEQYDEFFDVVRPMVLVQIANGMYSIITLIFLTL
LTYLSGYSIVSAPFLKFVCGLASLTIELYIYCYGFNHIEDGKSTVNFGLYSSNWT
EMDLKFKKTLLMAMVMNSAHKRVMKVSPNSIVNLEMFTGVMNMSYSIVSV
LLN

>MlytOR21
MENPQRVDRDSNGVTGNSAEKAEGGAEDDPAKDATDGGTILDVELFKIIGVY
QLLRPAEFGLNARVCRTTAIVVVCLTLGLQSMQVCRLYLARHDLQMFANVGV
MIINGLMCLLKGYMVAAYADRMSATLNSARYAFTGCGNRDQSKLRLCRARLS
TILRTFVRLSFGTLIVWVVMPWFMASEYDDTPTIWATVYVIESIILTVNVFCWT
SFDCYLVTMCFVLEALFCTMSTGYETLGRRRTGAKSSAPQQLGIEGTIINDVSI
SDVNYDDLTSHILDNQNIIEQYDEFFDVVRPMVLVQIANGMYSIITLIFLTLLVS
DTFLFPVRLGLWGGGWEDYD
>ApisOR21
MNNLSLRVSTSNDNDDNGVTENSAEKADDGTVENPAEKTAEGGTVLDVELF
RMIGVHQLLRPDEYGQDNELYRAVAKVIVGLTLVLQSMQVFRLYLARHDIMM
FAYIGVMIINGLMCLLKGYMVAAKADQMSATLTAAHYAFTKCGGRDPSKLRL
CRARLSAILRTFVGLSFGTLIVWLTMPWFMASDYDDQPFIWGVVYVIESIILTV
NVFCWTSFDCYLVTMCFVFEAQFCTMSTGYETLGRRQTGAKSSAPQKLGNA
STINNVKISDVNYEDLTSHIRDNQNIIKQYDAFFDVVRPMVLIQIANGMYSIIM
LIFLTLVTHLSGYSIFSAPILKFVCGLASLTIELYIYCYGFNHIEDGKSTVNFGLYS
SNWTEMDLKFKKTLLLAMTLNSAHKRVMKVSPNSIVNLEMFTGVMNMSYSI
VSVLLK
>SaveOR21
MGNPERADDNNGVTGNSAEKADGEAAENPPEKAADGDTVLDVELFQIIGVS
RLLRPDNSTMYRTAAKVVVGLTFGLQSMQVCRLYLARHDIPVFANIGVMFIN
GLMCLLKGYMVATNADQMRATLSAARYSFTKCGGRDPSKLRLCRARLSTILR
TFVGLSFGTLIVWLLMPWFMASDYDDRPFIWAAVYAAESIILTVNVFCWTSFD
CYLVTMCFVLEALFCTISTGYETLGRRRMGTKTSAHQQLGIAGTINNAKILSD
VNYDDLTSHILDNQNIIKQYDAFFDVVRPMVLIQIANGMFSIITLIFLTLLTHLS
GYSILSAPILKFVCGLLSLTIELYIYCYGFNHIEDGVRQKTPFHR
>ArumOR21
MENSRRTADGDRTVARNPAEKDDADGGTVLDVELFKSIGMYQLLRPAECGLN
VSRCRSAALGVVFMTLGLQSMQVCRLYLARNDFQMFANMAVLVINGLMCLL
KGYMVVANADRMCATLDAARYAFTGCGGRDPSELRRCRATLSAILRTFVALS
FGTLFVWVLIPWFMASEYDDMPTVWAVVYVVESTILTVNVFCWTSFDCYLVT
MCFVFDALFRTMSTGYETLGRCRSGDVKLSARQQFDTVAVADGEQYDAFFD
VVRPMVLIQIADGSYSIITLIFLTSLAYLKGYSIVSAPILKFVCGLASLTIELYIYC
YGFNHIEDGVKEKTSCHRSNIL
>AfabOR21
MANSRRTADGDRTVARNPAEKDDADGGTVLDVELFKSIGMYQLLRPAECGLN
VGRCRSAALGVVFMTLGLQSMQVCRLYLARNDFHMFANMAVLVVNGLMCL
LKGYMVVANADRMCATLDAARYAFTGCGGRDPSELRRCRATLSAILRTFVAL
SFGTLFVWVLIPWFMASEYDDMPTVWAVVYVVESTILTVNVFCWTSFDCYLV
TMCFVFDALFRTMSTGYKTLGRCRSGDVKLSARQQFDTVAVAGGEQYNAFF
DVVRPMVLIQIADGAYSIITLIFLTSLSYLKGYSIVSAPILKFVCGLASLTIELYIY
CYGFNHIEDGVKEKTSCHRSNIL

>AglyOR21
MANPRRTSDGDRTVARNPAEKDDANGGTVLDVELFKSIGMYQLLRPVECGLD
VGRCRSAALVVVFMTLGLQSMQVARLYLARHDFQMFANMAVLVINGLMCLL
KGYMVVANADRMCSTLDAARYAFTGCGGRDPSELRRCRATLSTILRTFVALSF
GTLFVWVLIPWFMASEYDDMPTVWAVVYVVESTILTVNVFCWTSFDCYLVT
MCFVFDALFRTMSTGYETLGRSRSGDVKLSARQQFDTVAAGAAISKTSITDDN
YDDLKSHIIDNKNIIEQYDAFFDVVRPMVLIQIADGSYSIITLIFLTSLAYLKGYS
IVSAPILKFVCGLASLTIELYIYCYGFNHIEDGKSTVNFGLYSSNWTEMDLKFK
KTLLLAMTMNSAHKRVMKISPNSIVNLEMFSRVMNMSYSIVSVLLNY
>RmaiOR21
MVNSRRTANDDRTTAPNPAEKDADGDTVMDVELFKSIGMYQLLRPAECGLNS
RRCRMAALVVVALTLGLQSMQVCRLYLARHDFQMFANMGVLVINGLMCLLK
GYMVVANADRMCATLEATRYAFTGCGGRDPSELRRCRATLSAILRTFVALSFG
TLFVWVLIPWFMASEYDDMPTVWAVVYVVESIILTVNVFCWTSFDCYLVTMC
FVFDALFRTMSTGYETLGRCRSSASKLSARQQFDTVGEQYDAFFDVVRPMVL
IQIADGSYSIITLIFLTSLAYIKGYSIVSAPILKFVCGLASLTIELYIYCYGFNHIED
GKSTVNFGLYSSNWTEMDLKFKKTLLLAMTMNSAHKRVMKVSPNSIVNLEM
FSRVIKLNVFQTRDQNFKNKIPTYLYVYTYIYTAGS
>AthaOR21
MENLRRTADGDRTVARNPAEKDDADGGTVLDVELFKSIGMYQLLRPAECGLY
VGRCRSAALAVVFMTLGLQSMQVCRLYLARNDFQMFANMAVLVINGLMCLL
KGYMVVANADRMCATLDAARYEFTGCGGRDPSKLRRCQATLSAILRTFVALS
FGTLVVWIIIPWFMASEYDDMPTVWAVVYVVESTILTVNVFCWTSFDCYLVT
MCFVFDALFRTMSTGYETLGRCRSGDVKLSVRQQSDTLLMYNILQLYFPTLPT
CRQYDAFFDVVRPMVLIQIADGSYSIITLIFLTSLVSDTSI
>ApisOR22
MKDILLNSIMHRNDSWIGSKIASSAALTNDGGGDEMTIHQDNSSNERDYQCE
DGGTAMDVKLFKAIGMYQLLHPVECGLNSDLCRKTAMMVVGLTVGLQLMQ
VFRLYLARHDIPMFANMAMLVVYGFMCLLKGYTLANHADRICITLEVARYAF
TDCGRRDPSLMRRCRARLSTILRTFVGLSFGTLVVWLVMPWFLASEYDGKPLI
WAVVYVVESIILTVNVFCWTSFDCYLVTMCFVFEAMFRTMSSGYEKVGRGQP
IHPHTNQPFGDHRRSDSEVKANVTLTFPSHYDDLISHIKDNQKIVEKYKTFFEI
VTPTVLLQIADGSYTIITMIFLISIAYLNGNSILSPMILKYVCGLVSLTIELYIFCYA
FNYIEDGRSTVNFGLYSCDWTDKDLKFKKTVLLAMSMNSANKQVMKLSPNS
IVNLEMFSRVMNMSYTIVSTLLS
>MvarOR22
MHRNGSWRRSKVASSAAPTDNGGDETTKNQDILSDEGDYDCKDGGTVMDV
DLFKAIGMYQLLHPVECGLDSGLCRMAVKIIVGLTLGLQSIQVCRLYLARYDIP
MFANMGVLVVYGLMCLFKGYTLAAHADRICTTLEVARYAFTSCGGRNPSLM
RQCRARLSTILRTFVGLSFSTLFVWLIIPWFLTSEYDDKPIVWAVVYVVESIIFT
VNVFFWTSFDCYLVTMCFVFEAVFRTMSYGYEKVGRGHHLHPHTFQPSGDY
RSIDSGEYVKLDVPLQFPDHYDDLINHIKDNQKIVEKYETFFEVVQPVVLLQIA
DGSYSVITLIFLISISYLNGNSIISPTILKFFCGLASLIIELYIFCYGFNHIEAGVKQ
KTESIK

>MperOR22
MRRSKIASSAAPTDNGGDEATKNQEISSDEGDYNCKDSGTIVDVDLFKAIGVY
QLLHPVECGLDSGLCRMAVKIIVGLTLGLQSIQVCRLYLARYDIPMFANMGVL
VVYGLMCLFKGYTLATHADRICTTLEVARYSFTSCGGRYPSLMRQCRARLSTI
LRTFVGLSFGTLFVWLIIPWFLTSEYDDKPIVWAVVYVVESIIFTVNVFCWTSF
DCYLVTMCFVFEAVFRTMSYGYENVGRVHRLYPHAVQPFLDYRSKDSDIQSD
VPLKFPDHYDDLINHIKDNQKIVEKYETFFDVVQPVVLLQIADGSYSVITLIFLI
SISYLNGDSIISPAILKFFCGLASVIIELFIFCYGFNHIEVGRSTVNFGLYCCDWTE
KDLKFKKTVLLAMSMNSAHKQVMKLSPNSIVNLEMFARVMNMSYTIVSTLL
S
>RpadOR22
MHAHRNRLKTASSTAASDDDAAAITLEIAGNEDDNYDREDGGTAVDVELFKT
IGIYQLLYPAECGLNAGRCRTAVLAVMCLTLGLQSMQVCRLYLARHDIQMFAN
MGMLVVYGFMCLLKGYTTTVNADRVSVTLDAARYAFTGCGGRDPSVMRRC
RATLSAILRTFVALSFGTLFVWVVIPWFIASEYDDMPTVWAVVYVVESFIFTVN
VFLWTSFDCYLVTMCFVFNALFRTMSAGYEKLGHRHRLLYPRHGHQPLHHSR
YPSEKYEIFFKIVRPVVLLQIANGSYSIITLIFLTSIAYLNGDSIVSPTIFKLVCALA
SLTIELYIYCYGFNHIEDGRSTVNFGLYSCNWTDKDLQFKKTLLLAMTINSAH
KLKMKVSPNSIVNLEMFTRV
>AcraOR22
MYQLLYPAECGLNDGRGYRTAMLAAMGLVLGLQSMQVFRLYLARHDIQMFA
NMGMLVVYGFMCLLKGHTTTTNASRMCVTLDAARYEFTGCGARDPSVMRR
CRTSLTAILRTFVALSFGTLVVWIIIPWFMASEYDDMPTVWAVVYVVESVIFTV
NVFLWTSFDCYLVTMCFVFDALFRTMSEGYEKLGHASRPYPHGRQPIRSHRG
QDTGVKYELFFQIVRPVVLLQIANGSYSIITLIFLTSIAYLNGDSIVSPAIFKLVCA
LISLTIELYIYCYGFNHIEDGRSTVNFGLYSCNWTDKDLQFKKTLLLAMTLNSA
HKLKMKVSPNSIVNLEMFTRV
>AfabOR22
MYQLLYPAECGLNDGSGYRTAMLAAMGLVLGLQSMQVYRLYLARHDIQMFA
NMGMLVVYGFMCLLKGHTTATNASRMCMTLDAARYEFTGCGGRDPSVMRR
CRTSLTAILRTFVALSFGTLVVWIIIPWFVASEYDDMPTVWAVVYVVESVIFTVN
VFLWTSFDCYLVTMCFVFDALFRTISAGYEKLGHASRPYPHGRQPLRSQRGQ
DTGEQYFFTSTSAVLSRKYELFFQIVRPVVLLQIANGSYSIITLIFLTSIAYLNGD
SIVSPAIFKLVCALISLTIELYIYCYGFNHIEDGVKQKSQFH
>AglyOR22
MYQLLYPAECGLNDGGDGYRTAVLAAMGLVLGLQSMQVCRLYLARHDIQMF
ANMGMLVVYGFMCLLKGHTTATNASRICVTLDAARYAFTGCGGRDPSVMRR
CRATLSTILRTFVALSFGTLVVWIIIPWFMASEYDDMPTVWAVVYVVESVIFTV
NVFLWTSFDCYLVTMCFVFDALFRTMSAGYEKLGHASRPYSHGRQPLRSHRG
QDTDVKRNLLKFPNDYDDLVSHIKDNQKIIEKYELFFQIVRPVVLLQIANGSYS
IITLIFLTSIAYLNGDSIVSPAIFKLVCALISLTIELYIYCYGFNHIEDGRSTVNFGL
YSCNWTDKDLQFKKTLLLAMTINSAHKLKMKVSPNSIVNLEMFTRVMNMSY
TIVSTLLS

>AgosOR22
MYQLLYPAECGLNDGGHGYRTAVLAAMGLVLGLQSMQVCRLYLARHDIQMF
ANMGMLVVYGFMCLLKGHTTATNASRICVTLDAARYAFTGCGGRDPSVMRR
CRATLSTILRTFVYDDMPTVWAVVYVVESIIFTVNVFLWTSFDCYLVTMCFVF
DMCFVFDALFRTMSAGYEKLG
>ApisOR20
MRSSSATVVDVMLFKAIGLYQLLCPADRGGYSVRSRRALMTALGLSFALHSF
QVPYLYYALNDLQRFAYMAAVIIYGMMCSFKGYVLVTNADRLWLVLNAADY
GYTGCGHRDPSRLRRCRATLSALLRTFVALSYGTLIVWIVLPFFVDEYTGITNS
DGTVTRYRTTIHNMQYPIPLAVYNSRPVWALIYVTELYVCIVNVFIWSLFDCYL
VTMCFVLNAQFHTMSAGYGTLGIRRTGSSPPDTTFAGVRRIKFDEIESNHYSD
LISHIQDNQNLIKMFDVFFEVVRPVVLVQIANGSYSVISLIFLTALMYLMGVPV
LSAAFLKFICGLISLTIELFIFCYGFNHIETAKSVLNFGIYSSNWTEMDLTFKKT
MLLTMKMNSSHKRAMKVSPNSAVGLEMFARVMNMSYSTVSVLLNSRS
>MvarOR20
MRSSSATAVDVMLFKTIGLYQLLCPADRGGYSVRFRRLLMTALGLSFALHSFQ
VPCLYYALNDLQRFAYMAAVIIYGMMCSFKGYVLVTNADRLWSVLDAAGYA
FTGCGHRDPSKLRRCRATLSALLRTFVALSYGTLIVWIALPFFVDEYTGVTNLD
GTVTRYRTTIHNMQFPVPLAVYNSRPFWALIYFTEVFVCIVNVFIWSLFDCYLV
TMCFVLNAQFHTMSAGYCTLGSRRAESSQPDTSRTGILYCYKTTVCYCLKDDI
VEFSIFTIQMYLMGVPVLSAPFLKFVCGLISLTIELFIFCYGFNHIETAKSVLNFG
LYNSNWTEMNLTFKKTMLLAMKMNSSHKRAMKVSPNSAVGLEMFARVSILIF
NLIFFFLKYVLLLTGA
>McerOR20
MRSSSATVVDVMLFKTIGLYQLLCPADRGGYSVRFRRLLMTALGLSFTLHAFQ
VPCLYYALNDLQRFAYMAAVIIYGMMCSFKGYVLVTNADRLWLALDVAGYA
FTGCGQRDPSKLRRCRATLSAVLRTFVVVSYGTLIVWIALPFFVDEYTGIINLD
GTVTRYRTTIHNMQFPVPLAVYNSRPFWALVYFTEVFVCIVNVFIWSLFDCYL
VTMCFVLNAQFHTMSAGYCTLGNRRAESSPPDTSLMGILYCYKKIFSIFTIQM
YLMGVPVLSAPFLKFVCGFISLTIELFIFCYGFNHIETAKSVVNLGLYNSNWTE
MDLTFKKTMLLAMGMNSSHKRAMKVSPTSAVGLEMFARVSIFILIN
>MperOR20
MRSSSATAVDVMLFKTIGLYQLLCPADRGGYSVRFRRLLMTALGLSFALHSFQ
VPCLYYALNDLQRFAYMAAVIIYGMMCSFKGYVLVTNADRLWLVLDAAGYA
FTGCGHRDPSKLRRCRATLSALLRTFVALSYGTLVVWIALPFFVDEYTGVTNL
DGTVTRYRTTIHNMQFPVPLTVYNSRPFWALIYFTEVFVCIVNVFIWSLFDCYL
VTMCFVLNAQFHTMSAGYCTLGSRRRRESSQPNTSRTGVRRIKFDDMESNHY
VDLIGHIQDNQKLIKVFDVFFEVVWPVVLVQIANGSYSVISLIFLTALMYLIGV
PVLSAPFFKFVCGLISLTIELFIFCYGFNHIETAKSILNFGLYNSNWTEMDLTFKK
TMLLAMKMNSSHKRAMKVSPNSAVGLEMFARVMNMSYSIVSVLLNSRS
>SmisOR20
MRSSSATVVDVMLFKAIGLYQLLCPADLGGYSVRFRRALMTALGLSFALHSFQ
VPFLYYALNDLQRFAYMAAVIIYGMMCSAADYGYTGCGHRDPSTLRRCRATL
SSLLRMFVALSYGTLIVWIALPFFVDEYTGVTNLDGTVTRYRTTIHNMQYPVP

LAVYNSRPVWALIYVTEVYVCIVNVFIWSLFDCYLVTMCFVLNAQFHTMSAG
YGTLGIHRAESSPPDTMFDIFFEVVRPVVLVQIANGSYSVISLIFLTALMYLMG
VPVLSAAFLKFICGFISLTIELFIFCYGFNHIETAVRQLHFFLPTMMVFIILISLILC
CPQKSVLNFGVYSSNWTEMDLTFKKTMLLTMKMNSSHKRAMKVSPNSAVGL
EMFARVGVTVLY
>SaveOR20
MRSSSATVVDVMLFKAIGLYQLLCPADRGGYSVRFRRALMTALGLSFALHSF
QVPFLYYALNDLQRFAYMAAVIIYGMMCSFKGYVLVANADRLWLVLSAADY
GYTGCGHRDPSTLRRCRATLSSLLRMFVALSYGTLIVWIALPFFVDEYTSVTN
LDGTVTRYRTTIHNMQYPVPLAVYNSRPVWALIYVTEVYMCIVNVFIWSLFDC
YLVTMCFVLNAQFHTMSAGYGTLGIHRTTESSPPDTSFAGILYCYKRLIILFLLL
NFTIGIFYFFTIQMYLMGVPVLSAAFLKFICGFISLTIELFIFCYGFNHIETAVRRL
HFFLPTMMVFIILISLILCCPQKSVLNFGVYSSNWTEMDLTFKKTMLLTMKMN
SSHKRAMKVSPNSAIGLEMFARVGVTVLY
>RpadOR20
MRSSASATAVDVTLFKTIGLYQLLCPANRGGYSARSRHALLTVLGLSFALHAF
QVPWLYYALNDLQRFAYMAAVIIYGMMCAFKGYVLVTNADRLWSVLDAAG
YAYTECGRRNPLWLRRCGVTLSAMLRSFVALSYATLIVWIALPFFVDEFTGIIN
LDGTVTRYRTTIHNMQFPVPLALYNSRPVWMLIYFTEVAVCIVNVFIWSMFDC
YLVTMCFVLNAQFHTMSAGYVTLGRRRTQSSPPQTPDTSVRMKLNDVESNH
YDDLIGHIQDNQKLIKFGLTIFCLFIIQMYLMGIPVLSAPFLKFICGVISLTIELFIF
CYGFNHIETAVKQFDLILY
>RmaiOR20
MRSPASATAVDETLFKTIGLHQLLCPANRGGYSACFRRALLIVLGLSFALHAFQ
VPWLYYALNDLQRFAYMAAVIIYGMMCAFKGYVLVTNADRLWSVLGAAGYA
YTECGRRDPSWLRRCGVTLSAMLRSFVALSYATLIVWIALPFFVDEFTAIINLD
GTVTRYRTTIHNMQFPVPLALYNSRPVWMLIYFTEVAVCIVNVFIWSMFDCYL
VTMCFVLNAQFHTMSAGYVTLGRRRARPSPPETPDTSVRMKLNDVESNHYD
DLIGHIQDNQKLIKLGLTIFCFFIIQMYLMGIPVLSAPFLKFICGVISLTIELFIFCY
GFNHIETAVRQFNLILPIIMMFVLNLVIKLCDLHRNL
>AglyOR20
MAHSPSTTVVDVSLFKTIGLHQLLCPVNRGGYSVRFRRALMTALGLSFALHA
FQVPWLYCALNDLQRFAYMAAVIIYGMMCAFKGYVLVTNSDRLWWTLDAA
GYGYTECGGRDPSALRRCRVTLSALLRSFVALSYATLIVWIVLPFFVDEYTPIT
NLDGTVTRYRTTIHNMQFPVPLSVYNSRPVWTLVYYVTEVSVCIVNVFIWSIFD
CYLVTMCFVLNAQFRTMSTGYVTLGRRRVKPLPQDTPVKDVRIKFNDVKSNH
YDDLIGHIEDNRKLIKAFDVFFDIVRPVVLVQIGNGSYSVISLIFLTSLMYLMGV
PVLSASFLKFICGVISLTLELFIFCYGFNHIETAKSNINFGLYSSNWTEMDLKFK
KTLLLAMKMNSSHKRVMKVSPNSSVGLEMFARVMNMSYSIVSVLLNSRS
>AfabOR20
MAHPSSITAVDVSLFKTIGLHQLLCPVNRGGYSVRSRRALMTALGLSFAVHAF
QVPWLYYALNDLQRFAYMAAVIIYGMMCAFKGYVLVTNSDRLWWVLDAAG
YGYTECGGRDPSALRRCRVTLSALLRSFVALSYATLIVWIALPFFVDEYTPITN
LDGTVTRYRTTIHNMQFPVPLSVYNSRPVWTLIYVTEVSVCIVNVFIWSIFDCY

LVTMCFVLNAQFRTMSTGYVTLGRRRVKPLPQDTPVKGVRIKFNDVKSNHYD
DLIGHIEDNRKLINFIIQMYLMGVPVLSAPFLKFICGVISLTLELYIFCYGFNHIE
TAVNI
>AgosOR20
MAHSSSTTVVDVSLFKTIGLHQLLCPVNRGGYSVRSRRALMAHSSSTTVVDV
SLFKTIGLHQLLCPVNRGGYSVRSRRALMTALGLSFALHAFQVPWLYCALND
LQRFAYMAAVIIYGMMCAFKGYVLVTNSDRLWWVLDAAGYGYTGCGGRDP
SALRRCRVTLSALLRSFVALSYATLIVWIVLPFFVDEYTPITNLDGTVTRYRTTI
HNMQFPVPLSVYNSRPVWTLIYVTEVSVCIVNVFIWSIFDCYLVTMCFVLNAQ
FRTMSTGYVTLGRRRVKPLPQDTPVKGVRIKFNDVKSNHYDDLIGHIEDNRK
LIKAFDVFFDVVRPVVLVQIGNGSYSVISLIFLTSLMYLMGVPVLSAPFLKFICG
VISLTLELFIFCYGFNHIETAKSNINFGLYSSNWTEMDLKFKKTLLLAMKMNSS
HKRVMKVSPKSSVGLEMFARVMNMSYSIVSVLLNSRS
>RmaiOR16
MTTTGRRATAVADRCGGGDDGETAADLTLFKAIGLHHMLKSGTAGRRFRAAY
VWFARLIVAVQLLQVAGLYASANDLRRFASMAVLAFNGLMCAFKGLVLANNA
DRLREVLDVARYRYTACGHRRPADMQRTGATVSALLRTFATVSYCTLAVWVI
TPLLADVAYVQIVHRDGTAAAYRATIDNTWLPGMSETVYNWPPVWAAIYAIE
VIMLTANVFNWMMFDCYLLTVCFVLNGQFRTLAVGYATIGSQRPLRWCSCVR
SRSGDPIVSFCSFAIYYKQLYLYYYLCIKNCLTLYTVKCIKKRAYRKYDDFFEIV
RPVVIIQVVSSSISIVGLILLVELLYSMGESITFGPVLRLICALLSLTMEFYIYCYS
FNYIEIAKCTLNFGLYSSNWTAMDLKFKKTLFMAMSMNSAHMKVMKLSPKY
IINLEMFTSVSI
>RpadOR16
MTTTDRRATAVADRCGGGGDDDETAADLTLFKAIGLHHMLESGTAGRRFRA
AYVWFTRLVVAVQLLQVAGLYASANDLRRFASMAVLAFNGLMCAFKGLVLA
NNADRLREVLDVARYRYTACGHRRPADMQRTGATVSALLRTFATVSYCTLAV
WVVTPLLADVAYVQIVHRDGTAAAYRATIDNTWLPGMSETVYNWPPVWAAI
YAVEVIMLTANVFNWMMFDCYLLTVCFVLNAQFRTLAVGYATIGRQRSLRWC
SCVRSRSGDPTVNLLYSMGESITFGPVLRLICGLLSLTMEFYIYCYSFNYIEIAK
CTLNFGLYSSNWTAMDLKFKKTLFLAMSMNSAHMKLMKLSPRYIINLKMFTS
VSI
>ApisOR16
MPVKANECGENETAVDLTLFKTIGLQRMLDPGPAGRRLRATYKWIACLIVTIQ
LMQVVGLYASVNDLQRFASLAVVAFNELTCSFKGILMITNADRMRAVLDVALY
RYTTCGHRQPANMRLTSATVSTLLRTFTMIGYCMLVVWIIAPLFTGVGYVQVE
HSDGTTGAYRKTIDNMWLPGMSETVYNWPPVWATIYVTEVVMMTVDMVIW
IMFDCYLITVCFVLNAQFRTLAAGYETIGSQRLTLQRGVDLEKSMGGKSDDG
DIDSLDYYEELIVHIKDNQNIIEKHDEFLEIVRPVVITQVVSSSISIVGLVFLIELL
YFMGEPFTFGPILRLIFGLISIIIQFYIYCYSFNYIEIAKCTLNFGLYSSNWTEMDL
KFKKTLFLGMSMNSTHMKVMKLSPKSIINLEMFAAVMKMSYSVVSVILNSIK
K

>SaveOR16
MPVIANECDDGETAVDLTLFKVIGLHRMLDPGATGRRLRASYKWIAYLTVTIQ
LMQVVGLYASVNDLQRLASLALVAFNALMCLFKGILMVTNADRMRAMLDVA
LYRYTTCGHRQPANMRLTSATVSTLLRTFAVISYCTLVVWIISPLFTGVGYVQIE
HNDGTAGAYRKTIDNMWLPGMSETVYNWPPVWTTIYATEMVMMTVDMVIW
IMFDCYLITVCFVLNAQFRTLAAGYETIGCQRLSLRPGVDFDKSMCGKSDDG
DIDSLNYYEELIDHIKDNQNIIEKHNEFFEIVRPVVITQVVSSSISIVGLVFLIQLL
YFMDEPLTFGPILRLIFALISIIFQYYFYCYSFNSIEIAKSTLNFGLYSSNWTEMD
LKFKKTLFLAMSMNSAHMKVMRLSPKSIINLEIFVAVSI
>SmisOR16
MPVIANECDDGETAVDLTLFKVIGLHRMLDPGATGRRLRASYKWIAYLTVTIQ
LMQVVGLYASVNDLQRLASLALVAFNALMCLFKGILMVTNADRMRAMLDVA
LYRYTTCGHRQPANMRLTSATVSTLLRTFAVISYCTLVMWIISPLFTGVGYVQIE
HNDGTAGAYRKTIDNMWLPGMSETVYNWLPVWTTIYATEMVMMTVDMVI
WIMFDCYLITVCFVLNAQFRTLAAGYETIGCQRLTLRPGVDFDKSMGGKSDD
GDIDSLDYYEELIDHIKDNQNIIEKHNEFFEIVRPVVITQVVSSSISIVGLVFLIQL
LYFMDEPLTFGPILRLIFALISIIFQYYFYCYSFNSIEIAKSTLNFGLYSSNWTEMD
LKFKKTLFLAMSMNSAHMKVMRLSPKSIINLEIFVAVSI
>ApisOR18
MTTPRVTAFTVPASSEDLTIVDNKLFKAICLHQILDPTNGGNRFCKLVLMAFMS
VSLSVQIMQLVGLYFAVNDLQRFAFTTTTLSYAFLCMTKDYVLLAHADRLRDS
LEVARFEFTSCGARDQRVVRRSRAVLSMVLRTFAMLSWSTCVIWALVPLFMM
DEYLQVTNADDTVSRYRVTIFNMWLPVPVAVYNATPIWSLIYMVEVIACLFTS
FSWLLFDSYVVTMCVTFNAQLRTVSASCATIGHRDCFASLSPHVTGTHIIKIDD
NSILSNCYDELIIHIKDNQNIIKKYDDFFEIIQPVVLFQIIAGSYSVITLIFLTALSY
LMGWSIISGPVLKVFFGFLSLTFELFLYCYVFNHIETEKCKMNFGLYSSNWTA
MDLKFKKTLLFAMNVNSAHRRVMKVTPTSIINLEMFANVMNMAYSIVSVLLN
SRVQK
>SmisOR18
MTTPRVTAFTAPASSEDLTIVDNKLFKAICLRQILDPTNGGNRFCRLVLMAFMF
VSLSVQIIQSIGLYFAVNDLQRFAFTTTTLSHAILCMTKDYVLLTHADKLRDSLE
VARYEFTPCGARDQRMVRQTHAVLSTVLRTFTVLSWVTCFIWALGPLFMMDE
YLQVTNIDGTVSRYRVTIFNMWLPVSVAVYNATPIWSLIYMVEVIACLFTSFSW
LLFDSYVVTMCVTFNAQLRTVSASCATIGHRDGSHRFASLSPHATGTNNIQIDD
NNLSNCYDELINHIKDNQNIIKKYDDFFKIIQPVVLFQIIGGSYSVITLIFLTVLA
YLMGWSILSGPVLKVFFGFLSLTFEFFLYCYVFNHIETEKCKMNFGLYSSNWT
VMDLKFKKALLFAMNMNSAHRRVMKVTPTSIINLEMFANVSTSYIN
>SaveOR18
MTTPRVTAFTAPASSEDLTIVDNKLFKAICLRQILDPTNGGNRFCRLVLMAFMF
VSLSVQIIQSIGLCFAVNDLQRFAFTTTTLSHAILCMTKDYVLLTHADKLRDSL
EVARYEFTPCGARDQRMVRQTRAVLSTVLRTFTVLSWVTCFIWALGPLFMMD
EYLQVTNIDGTVSRYRVTIFNMWLPVSVAVYNATPIWSLIYMVEVIACLFTSFS
WLLFDSYVVTMCVTFNAQLRTVSASCATIGHRDGSHRCHRTPRVRTNNIQIDD
NNLSNCYDELINHIKDNQNIIKKYDDFFKIIQPVVLFQIIGGSYSVITLIFLTVLA

YLMGWSILSGPVLKVFFGFLSLTFEFFLYCYVFNHIETEKCKMNFGLYSSNWT
VMDLKFKKALLFAMNMNSAHRRVMKVTPTSIINLEMFANVSTSYIN
>ApisOR17
MTTTPRVTELTAPASEDLTIVDNRLFKAICLHQILDPTKGGNRYYRLAFMVVM
WVSLSVQIIQLVGLYFAVNDLQRFAFTTTVIFNALLCLSKGYVLVVNADRLRAS
LEVARYEFTSCGARNQRLVRRSRAVLSTILRTFAVLSWVTCFIWALTPLFAMDE
YLQVTNADGTVSRYRVTIYNVWLPVPATVYNETTVWSLVYAVEVIACFVNVF
SWLLFDSYVVTMCFTFNAQFRTVSASTTIGHHSDSFRSPPPHAPEGTSDDNNT
FNCYDELINRIKDNQSIIKIYDDFFEILQPAILFQIIGGSYSVITLIFLTSLTYLMGF
SIISIPVLKVFFGFLSVTFELFLYCYVFNHIETEKCNMNFGLYSSNWTAMDLKF
KKTLLFAMNTNSSHRRVMKVTPMSIINLEMFANVMNMSYSIVSVLLNSRVQK
>SmisOR17
MTTTPRATEFTAPASEDLTVVDNMLFKAICLHQILDLTKGGNRYYQIGVRGGY
VMGVAKRADHTVGRPLLRSRRPTAVRVHYRDDIQRLALPVRGTQFTSCGARN
QRLMRRCRAVLSAVLRTFAVLSRATRFIWALTPPFPMDEYLQLTNADGTVSRY
RLQRVVTGAGNRVQPDGRLVARLRGRGDRVRRKRVQLVAVRQPRGDNVFNA
QFHTVTASCTTIGHRGVSFRSPSPHASEGTSDDNNTLNCYDELMNHIKDNQIL
KVFFGFLSVTFELFMYCYVFNHIETEVNKQKINYSNWILKLILFKKYLIEMLSI
FIDRNVIQILDCKLDCNGFKI
>SaveOR17
MTTTTPRVTEFTAPASEDLTVVDNMLFKAICLHQILDPTKGGNRYYQIGVRGG
YVMGVAKRADHTVGRPLLRSRRPTAVRVHCRDDIQRLALPVRGTRIGGERGQ
AARQPASGPGTSSRRAAVWLPVPVTVYNRTVVWSLVYLTSSGRGDRVRRKRV
QSVAVRQPRGDNVFNAQFHTATASCTTIGHRGVSFRSPSPHASEGTSDDNNTL
NCYDELMNHIKDNQILKVFFGFLSVTFELFMYCYVFNHIETEVNKQKINYSN
WILKLILFKKYLIEMLSIFIDRNVIQILDCKLDCNGFKI
>MperOR17
MTTTGATTFAPASEDLTIVDNKLFKAICLHQILDPTKGRNRYYRSALLAVMWL
SLSMQITQLVGLYFAVNDLQRFAFTTTTVTNAFQCLSKGYIIMTHADRLRASLE
TARYDFTSCGARDQRIVRRSRNVLSTVLRTFIVLSWVTCFIWALTPLFGMDEYL
QVTNADGTFTRYRVTIYNVWLPVPATVYNATAVWALVYSAEVIVCFVNVFSW
LLFDSYVVTMCFTFNAQFRTVSASCATIGHGDCSGSPSPHATGTHNIIRDDNNI
LNCYDELINHIKDNQSIIKKCDDFFEIIKPAILFQIIGGSYSVITLIFLTLLTYLMGF
SIISIPVLKVFFGFLSVTFELFMYCYVFNHIETEKCKMNFGMYSCNWTAMDLK
FKKTLLFAMNNNSAHRRVMKVTPKSIINLEMFSNVMNMSYSIVSVLLNSRVQ
K
>MvarOR17
MTTTGATAFTPASKDLTIVDNKLFKAICLHQILDPTKGGNRYYRTALLAVMWL
SLGVQITQLVGLYFAVNDLQRFAFTTTVVTNAFQCLSKGYIMVTHADRLRASL
ETTRYDFTSCGARDQRIVRRSRDVLSTVLRTFVVLSWVTCFIWALTPLFAMDE
YLQVTNADSTVTRYRVTIYNVWLPVPATVYNATPVWALVYSAEALVCFVNVF
SWLLFDSYVVTMCFTFNAQFRTVSASCATIGHRDCFGSPSPHATGSDDNNILN
CYDELINHIKDNQSIIKKCDDFFEIIKPAILFQIIGGSYSVITLIFLTSLTYLMGFSII
SIPVLKVFFGFLSVTFELFMYCYVFNHIETGKCKMNFGMYSCNWTVMDLKFK

KTLLSAMNNNSAHKRVMKVTPKSIINIEMFANVSIYIN
>MlytOR17
MTKTGATAFAPTSKNLTIVDNKLFKTICLHQILDPTKGGNRYYRSALLVVMWL
SFSLQITQLVGLYFAVNDLQRFAFITTTVINAFQGLSKGYVIVTLSDKLRASLEV
ARYDFTSCGASDQRIVRRSRNVLSTVLRTFVVLSWVTCFFWALTPLFTMDEYL
QLTNADHTVTRYRVTIYNVWLPVPATMFNATPVWALIYATEALVCFVNVFNW
LLFDSYVVTMCFTFNAQFRTVSASCATIGHRDCFGPSSPHATGSYLCDDFFEII
KPTILFQIIGGSYSVITLIYLTSLAYLMGFSILSVPVLKVFFGFLSMNFELFMYCY
VFNHIETEKSKINFGLYSCNWTAMDLKFKKTLLFAMNNNSGHRRVMKVTPMS
IVNLEMFANVSTYIN
>McerOR17
MTHSDKLRASLETARYDFTSCGAKDQHIVRRSRNVLSTVLRTFVVLSWVTCF
FWALTPLFAMDEYLQLTNADNTVTRYRVTIYNVWLPMPATMFNKMPVWALV
YSTEVLVCFVNVFNWLMFDSYVVTMCFTLNAQFRTVSASCATIGHHDCFGST
SPHATGSYLCDDFFEIIKPVIVFQIIGGSYSVITLIYLTSLVSILFFIICNMFHKQIL
KSNFHINLYQDVSYGFFNSIRTSFKSIFCCFMNFGLYSCNWTAMDIKFKKTLLF
AMNNNSGHRRVMKVSPKSIVNLEMFANVSTYVLY
>AthaOR17.1
MSTASSSEELTIVDNRLFKAIGLHQILNPTHGGNRYYRIALLAITWMSFVVQSM
QVVGLYFAVNDLQRFAFTTTMVSNGLLSISKTYVLGTNVDRLRDVLEAARYE
FRSCGSRDQRTVRRARTVLSTIVRTFTVLSYVLCFMWALDPLATIDEFLPVTNE
DGTVSRYRVTIFNVWLPVPVTVYNTTAVWAFLYAVEVIVCFFNVTSWLLFDSY
VLTMCFTFKAHFRTLSASYATIGHPDTFRSQTPHASGTYSYYRIYDDFFEVIKP
VILFQIIGGSYSVITLTFLTLMVNVSYGFFNYIHTCFKSIFGYLAVTFQLYLYCYV
LNLIETEKTTMNFGLYSSNWTAMNLKFKKTLLLAMNMNSAHRRVMKVSPKSI
INLEMFAKVCTYLYYLADNIIIVVHCFKVF
>AthaOR17.2
MSTASSSEELTIVDNRLFKAIGLHQILNPTLGGNRYYRIALLAITWMSFVVQSM
QVVGLYFAVNDLQRFAFTTTVVSNGLLSISKTYVLGTNVDRLRDVLEAARYEF
TSCGSRDQRTVRRVRTVLSRIVRTFTVLSYVTCFVWALDPLSTIDEFLPVTNAD
GTVSRYRVTIFNVWLPVPVTVYNTTAVWAFLYAVEVIVCFFNVTSWLLFDSYV
LTMCFTFKAHFRTLSASYATIGHPDTFRSQTPHASGTYSYYRIYDDFFEVIKPVI
LFQIIGGSYSVITLTFLTLMTYLMGFSIISTPVLKAFFGYLAVTFQLYLYCYVLNL
IETEKTTMNFGLYSSNWTAMNLKFKKTLLLAMNMNSAHRRVMKVSPKSIINL
EMFAKVCTYLYYLADNIIIVVHCFKVF
>AthaOR17.3
MSTASSSEELTIVDNRLFKAIGLHQILNSTHGGNRYYRIALLAITWMSFVVQSM
QVVGLYFAVNDLQRFAFTTTTMSNGLLSISKAYVLGTNVDRLRDVLEAARYEF
TSCGSRDQRTVRLTREVLSTIVRTFTVLSYVTCFMWALNPLSTIDEFLPVTNAD
GTVSRYRVTIYNLWLPVPVTVYNTTAVWAFLYAVEVIVCFFNVVSWLLFDSYV
LTMCFTFKAHFRTLSASYATIGHPDTFRSQTPHASGTYSYYRKYDDFFEVIKPV
ILFQIIGGAYSVITLTFLTSLTYLMGFSITSTPVLKAFFGFLAITFQLYLYCYVFNL
IETEKTAMNFGLYSSNWTAMNLKFKKTLLLAMNMNSAHRRVMKVSPKSIINL
EMFAKVCTYLYYLADNIIIVVHCFKVF

>AfabOR17
MATAPSSEELTIVDNRLFKAICLHQILNPTNGGNRYYRIALLAITWMSFVVQST
QLVGLYFAVNDLQRFAFTTTVMSNSFLSMSKAYVLATNVDRLRDGLEAARYE
FTSCGSRDQRTVRRARAALSTIVRTFTVLSYVTCFMWALNPLSAIGEFLPVTN
ADGTVSRYRVTIYNVWLPVPVTVYNTTAVWAVLYAVEVIVCFFNVISWLLFDS
YVLTMCFTFKAHFRTLSASYATIGHPDAFRSQTPHASGTYYTTTGTWKYDDFF
EVIKPVILFQIIGGSYTVITLIFLTSLTYLMGFSIISTPVLKAFFGFLAVTSQLYLYC
YVFNLIETEKTAVNFGLYSCNWTAMDIKFKKTLLLAMNMNSAHRRVMKVTP
KSIINLEMFAKVCTYL
>AcraOR17
MATASSSEELTIVDNRLFKAISLHQILNPTHGGNKYYRIALLAIMWISLVVQSM
QLVGLYFAVNDLQRFVFTTTVVSNSFLSMSKAYVLVTNVDRLRDGLEAAQYE
FTSCGSRDQRTVRRARAALSTIVRTFTVLSYVTCFMWAINPLSAIGEFLPVTNA
DGTVSRYRVTIYNVWLPVPVTVYNTTAVWAVLYAVEVIVCFFNVISWLLFDSY
VFTMCFTFKAHFSTLSASYATIGHPDALRSQTPHVSGTYYTTTGTWKYDDFFE
VIKPVILLQIIGGSYAVITLIFLTSLTYLMGFSIISTPVLKAFFGFLAVTFQLYLYCY
VFNLIETEVNKKQTT
>ArumOR17
MATAPSSEELTIVDNRLFKAICLHQILNPTHGGNRYYRIAILAITWMSFVVQST
QLVGLYFAVNDLQRFAFTTTVVSNSFLSMSKAYVLATKVDRLRDGLEAARYEF
TSCGSRDQRTVRRARAALSTIVRTFTVLSYVTCFMWALNPLSAIGEFLPVTNA
DGTVSRYRVTIYNMWLPVPVTVYNTTAVWAVLYAVEVIVCFFNVISWLLFDSY
MLTMCFTFKAHFRTLSASYATIGHPDAFRSQTLHASGTYYTTTGTWKYDDFFE
VIKPVILFQIIGGSYTVITLIFLTSLTYLMGFSIISIPVLKAFFGFLAVTFQLYLYCY
VFNLIETEKTAVNFGLYSSNWTGMDLKFKKTLLLAMNMNSVHRRVMKVTPK
SIINLEMFAKVCTYLYYLADNIIVVYCFKVF
>AglyOR17
MATAPPSEESTIVDNKLFKAICLHQILNPTHGGNRYYRIAILAILWMSIVVQITQ
LVGLYYAVNDLQRFAFTTTVVSNSFLSLSKAYVLVTNVDRLRDGLEAARYEFT
SCGSRDQRTVRRARAALSTIVRTFTVFSYVTCFFWILNPLSAIGEFLPLTNADG
TVSRYRVTIYNYWLPVSVTVYNTTTVWALTYAVEMIVCFFNVNTWLLFDSYV
LTMCFTFKAHFRTLSASYATIGHLDTFRSLTPHASSTYTGTWKYDEFFEVIKPVI
LFQMIGGSYTVITLTFLTSLTYLMGFSIISIPVSKAFFGFLVLTIQLYLYCYVFNHI
ETEKSAVNFGLYSSNWTAMDLKFKKTLLLAMNMNSAHRRVMKVIPRSIINLE
LFAKVCTYLYYLADNIIVVHCFKVF
>AgosOR17
MATASPSEEESTIVDNRLFKAICLHQILNPTHGSNRYYRIAILECIWMSIVVQIT
QLVGLYYAVNDLQRFAFTTTVVVNSFLSLAKAYVLMANVDRLRDGLEAARYE
FTSCGSRDQRTVRRARAALSTLVRTFTVFSYVTCFFWMLNPLSAIGEFLPMTN
ADGTVSHYRVTIYNYWLPVSATVYNTTTVWALTYAVEMTVCFFNVNTWLLF
DSYVLTMCFTFKAHFRTLSASYATIGHLDTFRSLTPHASGTKYDEFFEVIKPVIL
FQIIGGSYTVITLTFLTSLTYLMGFSIISIPVSKAFFGFLVLNFQLYLYCYVFNHIE
TEKSAVNFGLYSSNWTAMDLKFKKTLLLAMNMNSAHRRVMKVIPRSIINLEL
FAKV

>RpadOR17
MTTASEDLTIVDKRLFKAIGLFQILNPTPGANRCYRIAYMTSMIMSFVVQITQLI
GLYFAVNDLQRFAFTTTVISNGFLSLSKGFVLVTNADRLCAGLELARYEFTSCG
SRDQRTARGSRAVLSSILRMFLVLSSVTGFVWALTPLSLMGDYLPVTNMDGTI
SRYRVTIYNIWLPIPETVYNAPMVWALIYAVEVVVCFYNVYSWMLFESYVMT
MCFTLNAQFRILSDSFAAIGHRDQSPPPPHATGSKYDDFLEVIKPAVLLQIVSGS
YSVITLIFLTSLVGVLFFSKIMFHKLIFYFYFYFYLDVSYGCSNYIWTCFKIILWF
FVSYFSTIHKSAVNFGLYSSNWTALDLKFKKTLLLAMNMNSAHRRVMKVSPT
SIINLEMFANVSTYFIIIN
>RmaiOR17.1
MTTASEDLTIVDKRLFKAIGLHQILNPTPGANRYYRIAYMASMIMSFVVQIIQS
VGLYFAVNDLQKFAFTTTMISNALLCLSKGFVLVTNADRLRDGLEAARYEFTS
CGFRDQRAAHQARTALSTVLRTFVVLSYVTCFIWMLTPLSLMDDYLSVTNAD
GTVSRYRITIFNIWLPVPVTVYNAPTVWAFIYGVEMVVCFVNVFSWLLFDSYV
MTMRFTFNAQFRILSDSFAAIGHRDHSPPPTHTAGSCLAVYDDFFEVIKPAVLL
QIISGSYSVITLIFLTSLVSVLCFSKIMFHKLYLYFHFYLDVSYGISNHIRTCFKSI
LWLFVSYYSTIFKSAVNFGLYSSNWTTLDLKFKKTLLLAMTMNSAHRRVMKV
SPTSIINLEMFANVSTYFIIIN
>RmaiOR17.2
MTTASEDLTIVDNRLFKAICLHQILNPNNGGNRYYRIAFLASMILLLVVQIIQLV
GLYFAVNDLPRIAFTTTMVSNALLCLSKGFVLVTNADRLRDGLEAARYEFTSC
GFRDQRAAHQARTALSTVLRTFVVLSYVTCFIWMLTPLSLMDDYLSVTNADG
TVSRYRITIFNIWLPVPVTVYNAPTVWAFIYGVEMVVCFVNVFSWLLFDSYVI
TMCFTFSAQFRALSASFATIGHRRPLPTTIAARHKYELDEIMTMISTIISIFFFTYI
YRKYDDFFEVIKPAILLQIISGSYSVITLIFLTSLTYLMGFPIISGPVLKAFFGFLS
VTIQLFLYCYVFNYIETEKSAVNFGLYSSNWTTLDLKFKKTLLLAMTMNSAHR
RVMKVSPTSIINLEMFANVSTYFIIIN
