# Supplementary material for: Structural basis of sex pheromone detection in aphids
Source: Cell Res. 2026 Jun 22;36(8):582–94. doi: 10.1038/s41422-026-01267-z (PMC13424144; doi:10.1038/s41422-026-01267-z)
Supplement: Supplementary file 1 — Supplementary information, Fig. S1 [file 41422_2026_1267_MOESM1_ESM.pdf]

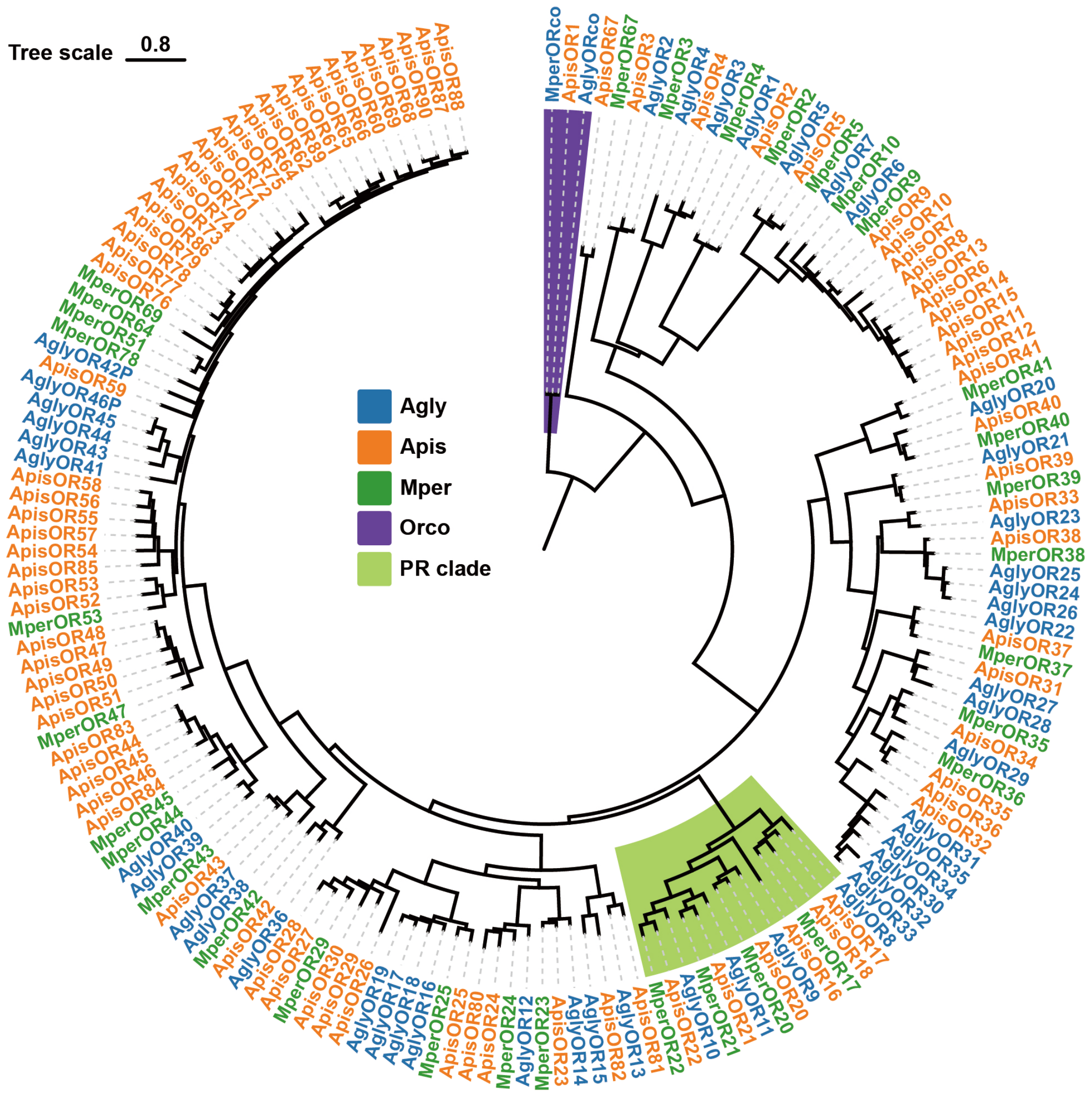

Supplementary information, Fig. S1 Phylogenetic analysis of odorant receptors from *Acyrthosiphon pisum*, *Aphis glycines*, and *Myzus persicae.* The maximum-likelihood phylogenetic tree was constructed using 1000 bootstrap replicates. A conserved clade of candidate sex pheromone receptors (*Ap*OR16/17/18/20/21/22) is highlighted.
