# Supplementary material for: Structural basis of sex pheromone detection in aphids
Source: Cell Res. 2026 Jun 22;36(8):582–94. doi: 10.1038/s41422-026-01267-z (PMC13424144; doi:10.1038/s41422-026-01267-z)
Supplement: Supplementary file 2 — Supplementary information, Fig. S2 [file 41422_2026_1267_MOESM2_ESM.pdf]

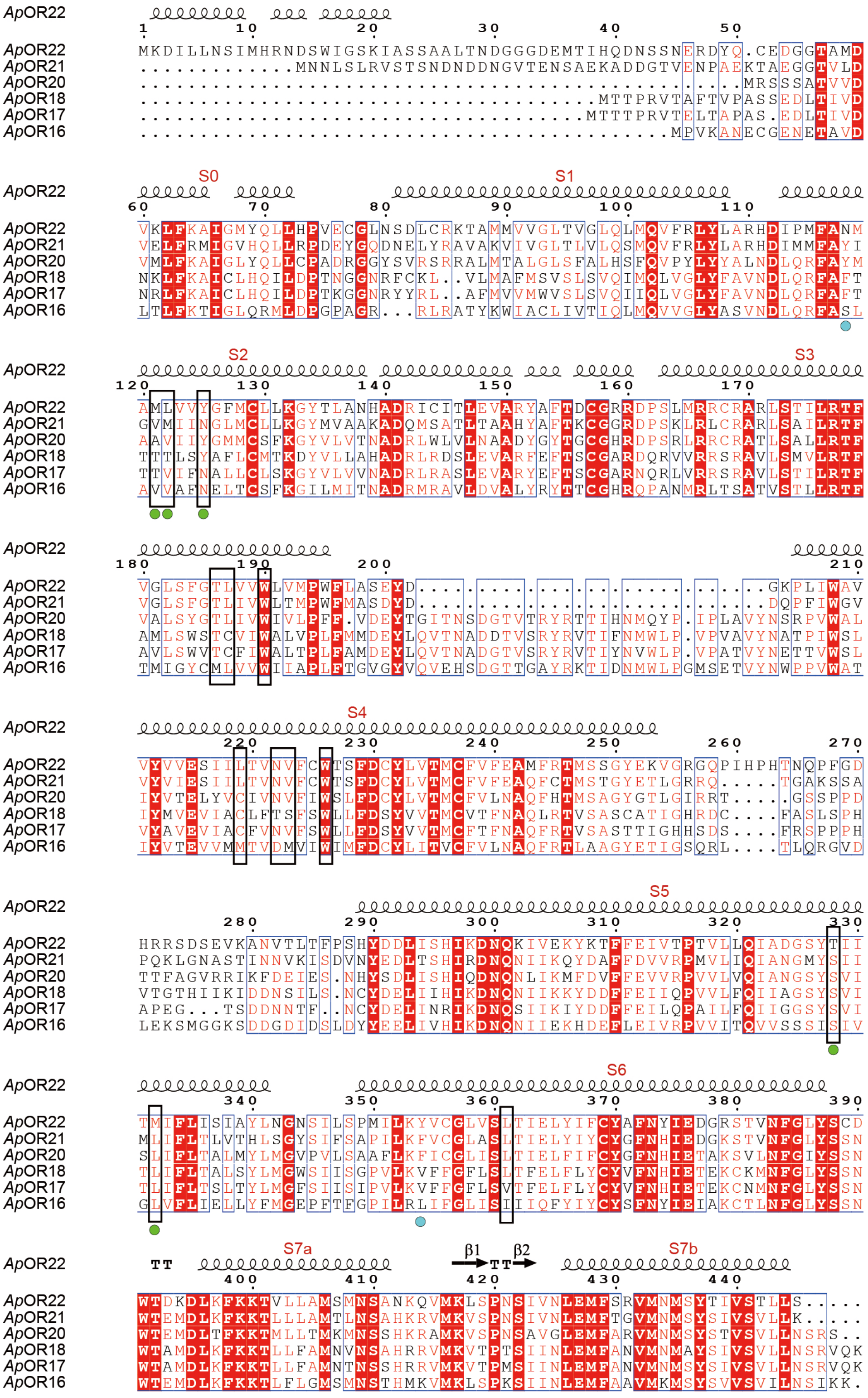

Supplementary information, Fig. S2 Sequence alignment of putative pea aphid PRs. Alignments were generated using MultAlin and ENDscript. Sequence identity is shaded red, similarity is marked in lighter red. Black boxes indicate 13 residues forming the nepetalactone-binding pocket of *Ap*OR22 and their corresponding sites in other ORs. Green circles denote amino acid variations between *Ap*OR22 and other receptors in the binding pocket. Blue circles indicate positions of N118 and Y354 in *Ap*OR22.
