# Supplementary material for: Structural basis of sex pheromone detection in aphids
Source: Cell Res. 2026 Jun 22;36(8):582–94. doi: 10.1038/s41422-026-01267-z (PMC13424144; doi:10.1038/s41422-026-01267-z)
Supplement: Supplementary file 7 — Supplementary information, Fig. S7 [file 41422_2026_1267_MOESM7_ESM.pdf]

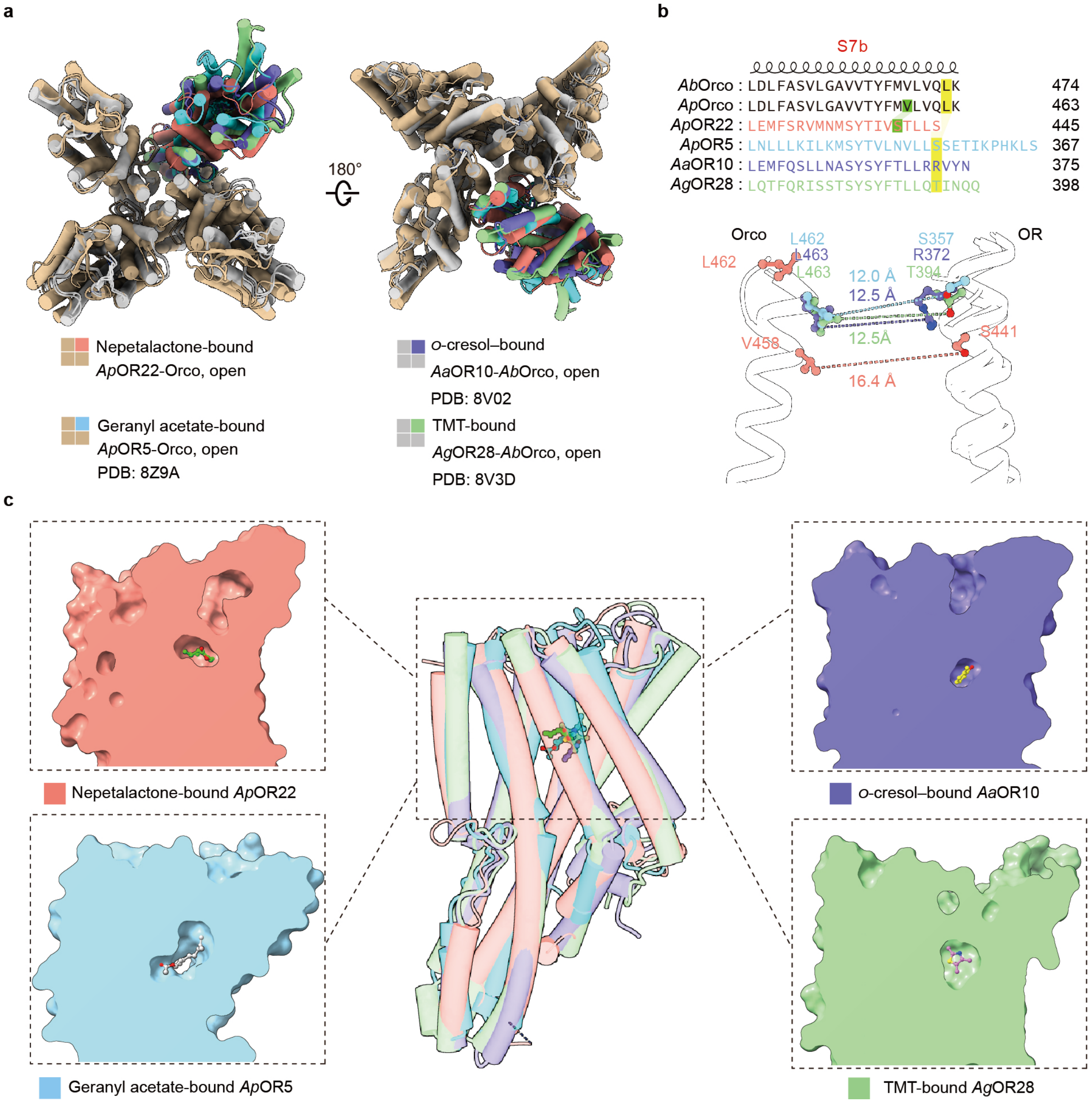

Supplementary information, Fig. S7 Structural comparison of *Ap*OR22-Orco with other OR-Orco complexes. a Structural comparison of nepetalactone-bound *Ap*OR22-Orco, geranyl acetate-bound *Ap*OR5-Orco, *o*-cresol-bound *Aa*OR10-*Ab*Orco, and TMT-bound *Ag*OR28-*Ab*Orco, shown in extracellular (left) and intracellular (right) views. **b** Sequence and structural alignment of S7b helices from *Ab*Orco, *Ap*Orco, *Ap*OR22 (salmon), *Ap*OR5 (light blue), *Aa*OR10 (light purple), and *Ag*OR28 (light green). Green and yellow shading mark residues forming the first layer adjacent to the pore. **c** Side views of ligand-binding pockets: nepetalactone in *Ap*OR22 (salmon, open state), geranyl acetate in *Ap*OR5 (light blue), *o*-cresol in *Aa*OR10 (light purple), and TMT in *Ag*OR28 (light green). Ligands are shown as ball–stick models: nepetalactone (lime), geranyl acetate (white), *o*-cresol (yellow), and TMT (violet).
